# The Right to Sexuality, Reproductive Health, and Found a Family for People with Intellectual Disability: A Systematic Review

**DOI:** 10.3390/ijerph20021587

**Published:** 2023-01-15

**Authors:** Patricia Pérez-Curiel, Eva Vicente, M. Lucía Morán, Laura E. Gómez

**Affiliations:** 1 Department of Psychology and Sociology, University of Zaragoza, C./Pedro Cerbuna, 12, 50009 Zaragoza, Spain; 2 Department of Education, University of Cantabria, Av./de los Castros, 52, 39005 Santander, Spain; 3 Department of Psychology, University of Oviedo, 33003 Oviedo, Spain

**Keywords:** Convention on the Rights of Persons with Disabilities (CRPD), intellectual disability, sexuality, fertility, parenthood, motherhood, sexual rights, sexual health, sex education

## Abstract

Although sexuality, reproductive health, and starting a family are human rights that should be guaranteed for all citizens, they are still taboo issues for people with intellectual disability (ID), and even more so for women with ID. This paper systematically reviews the current qualitative and quantitative evidence on the rights of people with ID in regard to Articles 23 (right to home and family) and 25 (health, specifically sexual and reproductive health) of the Convention on the Rights of Persons with Disabilities (CRPD). A systematic review of the current literature, following PRISMA 2020, was carried out in ERIC, PsychInfo, Scopus, PubMed, ProQuest, and Web of Science. In all, 151 articles were included for review. The studies were categorized into six themes: attitudes, intimate relationships, sexual and reproductive health, sexuality and sex education, pregnancy, and parenthood. There are still many barriers that prevent people with ID from fully exercising their right to sexuality, reproductive health, and parenthood, most notably communicative and attitudinal barriers. These findings underline the need to continue advancing the rights of people with ID, relying on Schalock and Verdurgo’s eight-dimensional quality of life model as the ideal conceptual framework for translating such abstract concepts into practice and policy.

## 1. Introduction

Since the adoption of the Convention on the Rights of Persons with Disabilities (CRPD) [1], the global commitment to equality, dignity, and freedom for people with disability, as adopted by the States Parties, has been apparent, creating a new discourse and causing a paradigm shift [2,3,4]. This paper focuses on the state of the art of two specific rights of the CRPD: home and family (Article 23) and sexual and reproductive health (Article 25). 

Article 23 (home and family) refers to the right to have opportunities to meet people, establish relationships, have friends, have a partner relationship and choose their sexual orientation; to marry and found a family, which includes retaining their fertility on an equal basis with others, making their own reproductive and sexual choices, and deciding the number of children to have; to keep their own children with them; to receive sexual information, guidance, and support and care for their children; to be able to adopt and foster and to access assisted reproduction; to access non-discriminatory support in sexuality; and to receive comprehensive sex education programs.

Article 25 (health) discusses the right to health, highlighting indicators and personal outcomes associated with good physical health (including sexual health); prevention; access to appropriate information on health-related issues; promotion of health behaviors in accessible formats; shared decision-making; supervised, justified and adjusted medication or medical treatment; and health screenings and being tested for sexually transmitted diseases. 

These above-mentioned CRPD articles (Articles 23 and 25) argue that sexuality is a central aspect of human functioning, present throughout life [5], that cannot be denied or compromised based on disability. They also establish the right of people with intellectual disability (ID) to make free, responsible, and non-discriminatory decisions regarding parenthood [6]. Sharing the CRPD’s principles and philosophy, the Sustainable Development Goals (SDGs), with their central promise to “leave no one behind”, define the actions that all countries must take to improve health and reduce inequality, recognizing that the inclusion of people with disabilities is fundamental to sustainable development [7]. As discussed by Gómez et al. [8], there is a close relationship and overlap between the SDGs and the CRPD Articles. In particular, Article 23 (respect for home and family) and Article 25 (health) of the CRPD fit with the SDGs of good health and wellbeing (SDG3), and Article 6 (women with disabilities) fits with the SDG on gender equality (SDG5). Likewise, several authors [2,3,8,9] have suggested that the quality of life (QOL) construct provides a valid framework from which to operationalize, measure, and implement the CRPD Articles [10].

The QOL model proposed by Schalock and Verdugo [11] is one of the most widely accepted and cross-culturally validated models of QOL [12], and it is used widely internationally in policy development, support provision, organization transformation, systems change, and outcome evaluation [13]. According to this conceptual framework, QOL encompasses eight core domains (rights, self-determination, social inclusion, interpersonal relationships, personal development, emotional wellbeing, material wellbeing, and physical wellbeing) that interact with each other and reflect the degree to which people have experiences that are valued to them. The QOL construct has recently been merged with the supports paradigm to produce the Quality of Life Supports Model (QOLSM), in which the QOL construct serves as a guide to obtain valuable information about *what* is important in a person’s life, while the supports paradigm provides guidance on *how* to achieve valued outcomes [2,3,4].

The right of all persons with disabilities to marry and start a family received its first serious discussion at the international level during the drafting of the Declaration of General and Special Rights of Mentally Retarded Persons in 1971 [14]. Sexual and reproductive health rights, for their part, are important for human happiness and wellbeing. Reproductive health addresses the broad determinants of women’s and men’s autonomy in reproductive decision-making and focuses on the legal, social, and ethical contexts in which these decisions are made [15].

There is no doubt that there have been important advances in the recognition of the rights of persons with disabilities in recent decades. However, despite the commitment of most countries to these rights, people with disabilities continue to experience multiple levels of discrimination [16], and people with ID, in particular [17], form one of the most vulnerable groups [18]. This statement provides the basis for the objectives and research questions of the present review.

This paper aims to synthesize the current qualitative and quantitative evidence related to the content of Articles 23 (home and family) and 25 (health, specifically sexual and reproductive health) of the CRPD, focusing exclusively on people with ID. The main research question focuses on the current status of persons with ID with respect to these two rights: Is the right to home and family and the right to the sexual and reproductive health of people with ID respected? In other words: (a) what are the attitudes toward the sexuality of people with ID?; (b) what barriers and facilitators do people with ID encounter in exercising their right to home and family?; and (c) what barriers and facilitators do people with ID encounter in exercising their sexual and reproductive rights?

## 2. Materials and Methods

The current review was conducted according to the Preferred Reporting Items for Systematic Reviews and Meta-Analyses (PRISMA-P) tool and reported in line with PRISMA guidelines [19]. 

### 2.1. Search Strategy

Potentially relevant studies were identified by an initial search and reference retrieval conducted by the first author in February 2022 using a single-line-search strategy based on free-text search terms in three relevant databases: Web of Science, ProQuest, and Scopus, which include PubMed, Elsevier, Education Resource Information Center (ERIC), and PsycInfo.

Search terms were introduced into six independent Boolean searches, as indicated in Table 1. The search strategy was limited to including these terms in English in the title, keywords, or abstract information. We filtered the results by selecting peer-reviewed publications and studies published in scientific journals.

### 2.2. Eligibility Criteria

All authors agreed on the eligibility criteria for studies to be included in the systematic review. These criteria are detailed in Table 2.

### 2.3. Study Selection

Following PRISMA guidelines and using the inclusion and exclusion criteria, studies were selected in three consecutive phases: (1) identification, (2) screening (divided into two phases), and (3) inclusion. 

After carrying out six independent searches in three databases, we identified 2784 records. A total of 281 duplicated records were eliminated before the screening phase, leaving 2503 articles eligible for inclusion. 

In the first phase of screening, all remaining records were checked by title and abstract according to the inclusion criteria: 2103 articles were eliminated because they were not related to the topic and aim of this review, were not empirical, or were not related to people with ID. To give the review greater validity, an interrater agreement was carried out in this phase. Two authors independently reviewed 30% of the entries (*n* = 750). One of the authors coded all the entries, and the other coded the 30%. Both results were compared, and the percentage of agreement was calculated to be 94.04%. In all, 400 articles passed this first cutoff. 

The second phase of screening involved a full-text review of the 400 references for further examination and eligibility determination. A total of 249 articles were excluded. A new interrater agreement was performed in this second phase, following the previous procedure. Again, 30% of the records (*n* = 121) were reviewed, this time reaching 90.75% agreement.

In both screening phases, agreements and disagreements were identified. Disagreements were discussed by jointly reviewing the inclusion and exclusion criteria and reaching a consensus. Where a consensus could not be reached, a third author was called in to make a final decision.

**Figure 1:**
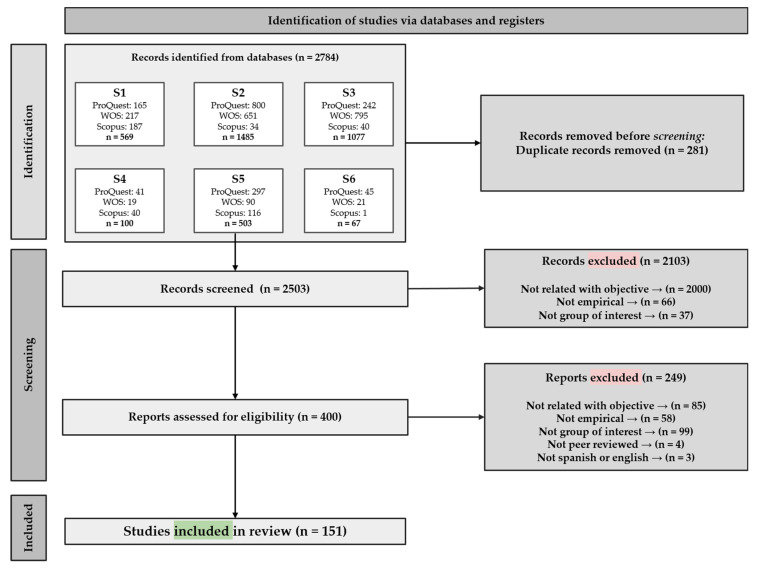
Flow diagram.

In the end, 151 articles—all written in English—were included for coding and analysis in the present review. The procedure is summarized in Figure 1. 

As mentioned previously, six independent searches were carried out. The final number of articles selected in each of these searches was: Search 1: Marriage (*n* = 24); Search 2: Family (*n* = 34); Search 3: Parenthood (*n* = 59); Search 4: Intimate relationships (*n* = 18); Search 5: Menstruation (*n* = 50); and Search 6: Sterilization (*n* = 11). Note that the total yield of the searches was greater than the total number of items because some items were returned by more than one search.

### 2.4. Synthesis Methods

The articles were described based on the following variables: (a) the objectives of the study; (b) localization; (c) characteristics of study participants; (c) type of study and design; and (d) main results. Given the large number of papers included, it was considered appropriate to categorize them in order to analyze them. From the categorization of study objectives and results, six major themes (and their respective subthemes) emerged (Table 3). These categories roughly aligned to the six searches carried out, although the same article could be classified under more than one thematic area, and the same article could be found within several subthemes of the same thematic area.

The Results section describes the articles included in the review, organized by theme and subtheme. A schematic synthesis of each of the six themes, including the most relevant information from each of the papers, is annexed as Supplementary Materials.

## 3. Results

### 3.1. Characteristics of Reviewed Studies

A total of 151 articles published between 1976 and January 2022 were included for review. A sizable number of articles (*n* = 22; 15%) were published in 2020 and 2021. Only eight of the papers (5%) were published before 2006, the year the CRPD was adopted. Bates, Brown, and McCarthy were the most prolific authors (*n* = 5 each). The 151 articles were published in a total of 84 scientific journals, with *Sexuality and Disability* featured as the main journal of choice. 

The studies were carried out across different geographical locations: 63 (42%) were conducted in Europe, with the United Kingdom (UK) in the lead (*n* = 30; 20%) followed by Sweden (*n* = 9); 49 studies (32%) were from America, of which 36 were conducted in the United States of America (USA), eight in Canada, and four in Mexico; 18 studies (12%) were carried out in Asia, with India and Nepal leading the investigations (*n* = 4 for each country); 13 studies (9%) were conducted in Africa, with South Africa leading the way (*n* = 4), followed by Uganda (*n* = 3); and 13 studies (9%) were from Oceania, with Australia being the most prolific (*n* = 11). With regard to methodology, 72 of the investigations were quantitative (47%), 68 were qualitative (45%), and 12 used mixed methods (8%). Most of the quantitative studies employed questionnaires (*n* = 62; 90%); four of these used the ASQ-ID scale developed by Cuskelly and Bryde [171], and two opted for the 64 vignettes proposed by Esterle et al. [172]. The remaining quantitative studies were based on national surveys or clinical registries, many of them having a cross-sectional or predefined cohort design. Regarding the details and characteristics of the samples, 113 studies surveyed participants with ID and 38 surveyed proxies only (healthcare workers, family members, and carers).

Concerning the participants with ID, the vast majority were women: 8 studies focused exclusively on women or adolescent girls, while three studies included male participants only. Of the 36 studies that included both male and female participants, male participants were the majority in 11 of them, female participants were the majority in 5 of them, and both genders participated equally in 20 of them. For the remaining six articles, whose participants were children or young people with ID, the gender was not specified. Most of the studies (*n* = 83; 55%) did not indicate the participants’ severity of ID. Of those that did specify this aspect (*n* = 33), almost all (*n* = 30; 90.90%) included participants with mild and/or moderate ID and much less frequently participants with severe (*n* = 9) or profound (*n* = 5) ID. Only one study focused exclusively on people with severe and profound ID [88]. A total of 92 studies specified the participants’ age range. Among these, almost all (*n* = 75; 81.52%) focused on adult life (the most frequent age range was 20–40 years old), but only 12 studies included participants above 60 years old. Conversely, 17 studies included younger participants (7–20 years old), with a greater participation of girls in 11 of them.

When the study focused on proxies, the most frequent roles or relationships with people with ID were family members, such as mothers or parents (*n* = 12); staff carers (*n* = 11); health service providers, such as general practitioners (GPs), OB–GYNs, midwives, or social workers (*n* = 10); and teachers (*n* = 5). 

Further, 36% of the studies (*n =* 55) compared the participants with ID with other participants with other types of disability (e.g., physical disabilities, hearing and/or visual impairment, autism spectrum disorder, mental health illnesses) or without disability.

Of all the articles, 75% (*n* = 113) obtained their data directly from people with ID. Of this 75%, 19 studies also included the participation of proxies who acted as informants on behalf of the people with ID and completed or complemented the information provided by them. These proxies–informants were mainly primary caregivers (i.e., family members), staff, carers, teachers, and health service providers (i.e., GPs and midwives). 

### 3.2. Attitudes toward the Sexuality of People with ID

Of the 17 studies that dealt with this aspect, 10 studies relied on quantitative methods, with 5 studies using the ASQ-ID and ASQ-GP questionnaires [171] and 2 studies using the 64 vignettes designed by Esterle et al. [172]. With the exception of one study, all had been conducted and published after 2010.

Only three studies included participants with ID. Participants in the remaining studies were healthcare personnel (*n* = 3), family members (*n* = 4), caregivers (*n* = 5), students (*n* = 2), and the general population (*n* = 2). 

The results were grouped into further subthemes according to the topic that participants were giving their opinion on: sexual freedom, sexuality, marriage, sterilization, and parenthood. A common thread running through many studies was the recognition of the impact of religion, culture, and gender on attitudes toward the sexuality of people with ID [22,23].

#### 3.2.1. Attitudes toward Sexual Freedom

Sexual freedom refers to the sexual and reproductive rights recognized by the IPPF International Planned Parenthood Federation Declaration [167] and encompasses the opportunity for individuals to have control and decide freely on matters related to sexuality.

The majority of studies reflected a negative attitude toward sexual freedom for people with ID compared with people without ID. It was generally seen as more acceptable for women with ID since men were considered to have less control over their impulses and sexual behaviors [23,24].

#### 3.2.2. Attitudes toward Sexuality

Attitudes toward the sexuality of people with ID were conservative [36], and talking openly about sexuality was a taboo subject, even more so when talking about LGBT people with ID, for whom the door seemed to be “definitely closed” [22]. 

Among the families of people with ID, there was considerable heterogeneity in opinions [28]. Participants expressed sympathetic views in some of the studies [23,24,34,35], acknowledging that sexuality is a right for all people and that it is a “human need to feel loved emotionally and physically” [22], although it was recognized that women with ID face numerous social obstacles and cultural taboos. However, for other participants, the sexuality of people with ID elicited negative attitudes [20,32]. Some of them reported that it was unnecessary to attend to the sexuality of people with ID, whom they regarded as asexual (or, on the contrary, hypersexual) and having no interest in sexual activity, with the added caution that sex education could awaken the very behaviors to be limited [29].

#### 3.2.3. Attitudes toward Marriage

Even though marriage is central to the faith and culture of many people, a large number of participants opposed marriage for people with ID. This resistance was related not only to the abilities of people with ID but also to the consequences of marriage, ranging from an increased risk of abuse (especially for women with ID) to two people with ID marrying and placing a double burden on families [26]. 

That said, some family members, especially mothers, wanted their children with ID to marry and believed that marriage was a source of peace of mind for them and their children as a guarantee of future care [34].

#### 3.2.4. Attitudes toward Sterilization

In all articles related to this topic, attitudes in favor of sterilization were highlighted, especially among physicians, older physicians in particular [24]. Considering disability as an irrevocable biological phenomenon, these informants neglected the biological and psychosocial dimensions of people with ID and considered sterilization to be an acceptable method of blocking fertility [32]. Physicians stated various situations when this practice would be recommended: when parenting presents a significant psychological health risk; when the individual is in a long-term relationship; when the individual resides long-term with family; or when there is a risk that the condition could be passed on to a child.

#### 3.2.5. Attitudes toward Parenthood

Relatives’ misconceptions led to the view that people with ID do not need affectionate or intimate relationships and love, and therefore, they are not suitable candidates for marriage or parenthood. Some also regarded the grandchildren as a burden on the family because they felt that their children with ID were incapable of caring for others [26,29].

Students in disability-related undergraduate courses showed more positive attitudes toward disability parenthood, with greater acceptance among younger students [25]. Staff workers, however, were less supportive of parenting for people with ID [23,35]. In their view, even if people with ID had the skills to be good parents, they could become overwhelmed by the difficulties of parenting. Doctors [24,34] highlighted some reasons why they were against people with ID becoming parents: society bears significant and ongoing costs in supporting their parenting role; there are limited services available to support the individual in their parenting role; the individual is vulnerable to sexual abuse; the individual has demonstrated ongoing hypersexuality or inappropriate sexual behavior; the individual is not considered to have the ability to raise a child (even with support); or the individual is unable to manage sexual hygiene.

Among the general population, there were a variety of attitudes, with the majority valuing parenthood for people with ID as long as certain conditions were met: a long-term relationship, a partner without ID, and support from their families [30].

### 3.3. Intimate Relationships

All articles categorized into this theme (*n* = 30) were published from 2006 onwards. Most (*n* = 24) included participants with ID, and in 18 of them, this population represented 100% of the sample. In the majority of studies, the participants were over 18 years old. In 17 articles (56.6%), participants of both genders were included. Only six studies (20%) specified the participants’ level of ID: the most frequent level was mild-to-moderate, while only one study included people with severe ID and profound ID. 

Four subthemes were identified: (a) desires and expectations; (b) barriers and facilitators; (c) marriage; (d) violence and abuse; and (e) interventions.

#### 3.3.1. Desires and Expectations

Seven articles collected information on the desires and expectations that people with ID have about affective and intimate relationships [37,38,39,40,41,42,43]. These studies were all qualitative and included males and females between 16 and 60 years old.

The participants suggested that up to 50% of people with ID were chronically lonely, despite people with ID recognizing love as an essential element for their wellbeing [41]. Being surrounded by people was perceived as meaningful, and love was even defined as a prerequisite for a good life. It was also considered to be every human being’s right. When thinking about the future, all participants hoped they would have love in their lives [42]. 

Romantic relationships were highly significant to women as they functioned as a productive nexus for understanding gender in their lives. Developing their roles—or aspirations—as “girlfriend”, “fiancée”, “bride”, and “wife” positioned the women as desirable, capable, and lovable [43]. Having a partner suggested a sense of normality and being an ordinary member of society [38].

#### 3.3.2. Barriers and Facilitators

Sixteen of the articles on this theme identified barriers or facilitators that people with ID encounter when initiating or maintaining intimate and affective relationships with other people [22,37,41,44,45,46,47,48,49,50,51,52,53,54,55,56]. Eleven studies included only people with ID as participants, while five focused on the perceptions of carers (either family or staff). One reason people with ID found it hard to have relationships was because, in general, they felt they were treated like children [37]. Support workers stated that they did not feel that people with ID were truly treated with the same human rights as everyone else: “It is still the old culture rippling on. It is all about risk” [45]. In order to support the human rights of this population, stopping the perpetuation of this stereotype was pointed out as an urgent need [50].

Barriers identified by people with ID included lack of understanding; vulnerability to abuse and organizational barriers such as restrictive attitudes and behaviors by support staff and relatives; lack of guidance and support from families and staff; and societal beliefs and attitudes (e.g., asexual, childlike, vulnerable to abuse). 

The environment seemed to exert a considerable negative influence. Parents wanted to safeguard the wellbeing of their children in a hostile world, although this mindset would hold back adult children with ID from being included in society, in order to keep them safe from harm [48]. Support workers emphasized the lack of support and guidance from organizations, which made them reluctant to take any risks and, thus, limited the support they could provide to people with ID [45,52]. They reported that policy guidance in this area was “unclear” and “restrictive” and identified with feelings of being “unsupported” and “frustrated” when working with the issues of sexuality and intimate relationships. Staff spoke of their internal conflict of wanting to protect the individuals they supported while also promoting their autonomy [22].

Professional support and family support for relationships were mentioned as facilitators [41,46]. 

#### 3.3.3. Marriage

Five articles dealt with marriage and people with ID [57,58,59,60,61]. Three of these five articles included participants with ID: one study covered all degrees of severity, and two studies compared the results with people without ID.

The marriage themes dealt with in the articles fell into two subcategories: (a) visions and beliefs about the right to marry for people with ID [59,60,61], and (b) concerns about forced marriages [57,58].

The marital status and housing profiles of adults with ID differed considerably from the general population [59]. Women with ID encountered many social barriers [60] and were more than twice as likely as women without ID to report being single (i.e., separated, divorced, or widowed). Half of the women with ID were single in their fifties in a population-based analysis conducted in Canada [61].

As for forced marriages, there was a lack of understanding among frontline workers and commissioners. Adequate guidelines and materials were often not incorporated into local government and national health service policies and strategic plans [57]. The overall risk of forced marriage was higher for people with ID, especially in particular cultural contexts [57,60]. Parents tended to support the marriage of daughters with ID as a means of alternative caregiving as they needed to make plans for the future life of their daughters [60]. Around half of all forced marriages took place when the victim was aged between 16 and 21. Although forced marriage is considered an issue that predominantly affects young women, recent data suggest that men with ID are equally likely to be forced to marry [58].

#### 3.3.4. Violence and Abuse

Five articles were classified within this subtheme [60,62,63,64,65]. All of them used a qualitative methodology and included participants with ID, and the results were compared with people without ID or with other disabilities. The articles discussed the violence and abuse perpetrated by and experienced by people with ID. 

From an ecological systems perspective, multiple risk factors across different contexts influenced the prevalence of dating violence for adolescents. Experiencing maltreatment was one of the strongest predictors of victimization and violence perpetration in dating relationships. Adolescents with borderline-to-mild ID were found to have access to fewer resources for developing healthy relationships and, therefore, might be more susceptible to violence and abuse, either as the perpetrator or the victim. When compared with youths without ID, a greater percentage of youths with borderline-to-mild ID had threatened to hit their partner (16% vs. 6%), had thrown something at their partner (28% vs. 7%), or had a partner that had pushed, shoved, or shaken them (29% vs. 7%) and had threatened them in an attempt to have sex (12% vs. 1%) [64].

Further, social attitudes and behaviors that viewed women as weak and dependent revealed the unequal power relations between men and women, rendering women more vulnerable to violence. About half of the women with ID had experienced sexual abuse or harassment [62]. 

People with ID were also more likely to report non-partner physical and sexual violence experiences. For all women with ID, parents and other family members were the main perpetrators of physical violence. Strangers were the main perpetrators of both physical and sexual violence against men with ID [63].

The absence of intimacy was not the only problem encountered by women with ID. Inadequate sex education and social services, insufficient family supervision, and an unbalanced marriage structure increased the risks of sexual abuse for women with ID. Therefore, ironically, marriage not only increased the risk of sexual abuse for women with ID but also made it legitimate and imperceptible [60].

Finally, one study [65] reported on an intervention called the *Friendships Dating Program for Adults with IDD* (FDP), based on the idea that safety training alone is not enough to prevent interpersonal violence. Participants were given a questionnaire at different time points (before the intervention, just after the intervention, and 10 weeks after the intervention). It was observed that the FDP had the potential to increase the size of social networks and reduce interpersonal violence. This study also demonstrated the community’s capacity to provide evidence-based services that help people with ID to develop healthy, safe relationships.

### 3.4. Sexual and Reproductive Health (SRH)

Of the 47 articles within this theme, a considerable number were published in the USA (*n* = 14) and the UK (*n* = 8). Almost half (*n* = 23) used a qualitative methodology. Only five were published before 2006, while most (*n* = 40; 87%) were conducted in the last decade.

The vast majority of the studies (*n* = 35) included participants with ID, of which 15 compared results with people without ID or with other disabilities. Regarding the characteristics of the participants with ID, the participation of women was much more frequent (23 articles included female participants only), with the most common age range between 20 and 40 years old. Six articles reported on participants with severe ID and four reported on people with profound ID, making this the theme with the highest number of participants with this level of severity.

Five subthemes were identified: (a) barriers and facilitators; (b) menstrual health management (MHM); (c) contraceptive choices; (d) sterilization; (e) human immunodeficiency virus (HIV) and sexually transmitted infections (STIs).

#### 3.4.1. Barriers and Facilitators

Most of the articles (*n* = 23) fell into this subtheme. Fourteen studies included participants with ID (they were the only participants in eight of them). The remaining nine articles involved caregivers or healthcare personnel (GPs, OB–GYNs, or midwives). Therefore, when compiling a list of barriers and facilitators, a distinction can be made between those encountered by people with ID themselves in their access to SRH and those encountered by caregivers or support workers.

##### Barriers and Facilitators Encountered by People with ID

Many participants perceived that the disadvantages faced by women with ID were not just because of their impairment but were due to the intersection of discrimination based on gender and disability [73]. Results showed that SRH services did not respond well in terms of the 6 A’s—approachability, acceptability, availability, accommodation, affordability, and appropriateness—reflecting the social dynamics [77]. Barriers can be classified as socioeconomic, structural, and attitudinal [79,84]. Socioeconomic barriers encompassed a lack of empowerment, a lack of family support, and cultural and religious factors. 

Structural barriers comprised long distances to health facilities and the inaccessibility of buildings (e.g., poor condition of pavements around health facilities, no access ramps, no automatic doors).

Attitudinal barriers were wide-ranging and included (a) negative and judgmental attitudes toward sexual activity; (b) the social acceptability of service users: in the participants’ narratives, disability intersected with age, pregnancy, marital status, educational status, and impairment type; (c) interpersonal characteristics of the providers: studies highlighted that people were cared for not as “person first” but rather as “disability first”; stigmatization and unprofessional behaviors from healthcare providers; perception that SRH was necessary for married people only; (d) perceptions of gendered barriers to the National Disability Insurance Scheme (NDIS) related to motherhood, childcare, and other caring responsibilities; (e) male GPs were often cited as barriers; and (f) GPs often communicated in ways participants could not understand.

From the perspective of the ecological model, some studies have proposed outcomes that would facilitate access to SRH. At the micro level, the facilitators consist of education opportunities and community participation in awareness-raising activities. At the meso level, they concern family members and service providers being trained on the diversity of experiences of people with disabilities and SRH rights, coupled with better accessibility of basic infrastructure as well as information and services. At the macro level, the key facilitator cited was the need to move beyond a policy on “paper” toward the implementation of tangible measures [76]. Other relevant enablers included good relationships with the surrounding community and family members to enable access, the interpersonal characteristics of the service providers, and the use of trained service providers to enhance respectful communication skills [81].

Barriers and Facilitators Encountered by Carers and Health Service Providers 

Among the challenges encountered by professionals, communication barriers stand out above all, as well as the feeling of not having sufficient training to attend to the needs of women with ID adequately [67,71,75,82].

Despite these barriers, many health professionals (especially midwives) expressed their desire to provide the best care and, to do so, the following strategies were suggested: (a) focusing on building a relationship with the women and gaining their trust; (b) never questioning the fact that women with ID are pregnant or want to become mothers; (c) maintaining a positive attitude toward women with ID while acknowledging their additional needs [67]; (d) extending the appointment time to ensure that the women receive optimal information and support and gain a feeling of trust and security; (e) adapting individual counseling according to the women’s abilities and needs; (f) using pedagogical methods, such as clear, simple words and questions, repetition, short sentences, pictures, models, easy-to-read brochures, and a recap of what was said [71]; (g) providing adequate information when the person with ID is mature enough to discuss the topic and repeating the information as much as needed; and (h) providing individuals with ID with practical knowledge and skills, using plain language and providing practical examples [35]. 

#### 3.4.2. Menstrual Health Management (MHM)

Eight articles dealt with MHM [87,88,89,90,91,92,93,94]; all involved the experiences of women with ID. In seven studies, the participants were women or adolescents with ID, with the degree of severity specified in three of them. In four of the studies, the information on the MHM of participants with ID was obtained from other informants, usually mothers. In contrast to previous themes, the age range was between 11 and 19 years. 

MHM challenges faced by caregivers included the inability to communicate about the start of menstruation, the refusal to wear sanitary napkins, and not maintaining personal and menstrual hygiene [90]. The majority of parents/guardians did not receive adequate information regarding the menstrual care of girls with ID [91]. Parents did not have sufficient knowledge and skills to manage the sexual behaviors of the adolescents, and they showed no interest in receiving education in this regard [87]. Some of the mothers believed that educating adolescent girls on their genital health would increase their sexual motivation. 

The inability to manage menstruation by adolescents with ID was statistically significantly related to the degree of ID [90]: Girls with mild ID were independent; those with moderate ID required supervision and training for independent menstrual care; and women with severe ID were fully dependent on their caregivers for maintaining menstrual hygiene [89].

#### 3.4.3. Contraceptive Choices

We identified 13 studies within this subtheme [35,69,70,71,82,84,95,96,97,98,99,100,101]. Nearly half (*n* = 6) were conducted in the UK. Nine articles included participants with ID (in seven, they represented 100% of the sample) and three involved other important actors in the lives of people with ID, such as workers, midwives, or family planning consultants. In the nine studies involving people with ID, all participants were women with ID, but only four studies specified the level of ID, with mild ID being the most common. Two articles included participants with severe ID and one with profound ID.

Most participants indicated they had varying levels of control regarding their sexuality and reproductive health decisions [70]. The most remarkable feature of the participants’ responses regarding their use of contraception was a lack of autonomy. The vast majority of the women had not been given any accessible information about contraception. Moreover, for most of them, it was the norm to be accompanied to medical appointments by staff or their mothers [97]. Consequently, they felt that important decisions about their contraceptive use were largely made by others, mainly by GPs, staff in disability services, and parents [95,96,100,101].

Staff agreed that the initiative to discuss contraception often came from parents and staff, more rarely from the individuals themselves, especially if they had a more severe level of ID [35]. Caregivers, including family members and residential facility staff, played an important role in contraceptive selection and access [101]. Culture, religion, and the woman’s financial situation played a significant role in contraceptive choices [70].

Women with ID usually began to take contraception when they were not sexually active, and decisions were made on two bases, either to manage menstruation or to avoid pregnancy risk (related to fear of abuse) [84,95]. By far, the most widely used form of contraception was the contraceptive implant, commonly known as the Depo-Provera injection, followed by the pill and the IUD [97,101]. Not having to handle tablets made things easier for caregivers [35].

#### 3.4.4. Sterilization

Seven studies were included in this subtheme [21,62,66,102,103,104,105]. All participants were women with ID, with an average age between 20 and 30. Four studies included participants with ID, although, in two of them, they were part of a larger sample of women with other disabilities or without disabilities. The remaining three studies involved other stakeholders, such as doctors, family members, or support staff from specialized centers.

Women with ID had the greatest risk of undergoing sterilization. The results suggested that nontherapeutic hysterectomy continues to be a common procedure performed in females with ID [104,105]. OB–GYNs expressed strong views regarding routine contraception and sterilization for women with ID [66], indicating that the procedure might be warranted for both men and women with ID when [102] (a) it is in the best interests of the person; (b) the person cannot raise a child; (c) the person lacks the capacity for reproductive decision-making; (d) the person is incapable of giving informed consent; or (e) the person cannot provide valid consent to a marriage contract.

The consequences of a hysterectomy were usually explained to the parents in terms of “better quality of life” and as a safe surgical procedure [105]. Sterilizations were also justified as a way to prevent sexual abuse, even though, in reality, the procedure only prevents pregnancy, not abuse [62]. Women with ID who were more likely to be sterilized were younger had less education, were on a low income, and were not married [103].

#### 3.4.5. HIV and STIs

Three of the four studies in this subtheme were carried out on the African continent and were based on quantitative methods. All studies included males and females with ID, together with people with other disabilities (*n* = 2) or without ID (*n* = 2). Only one study specified the level of ID (i.e., mild to moderate), and the age ranged from 12 to 64 years.

Having ID was significantly associated with lower levels of knowledge about HIV transmission. This was related to inadequate exposure to HIV information at home and school because parents and teachers often lacked the expertise to give such information or had the fear that it would encourage promiscuity. People with ID believed that HIV could be transmitted through kissing or even sharing a toilet or cup. However, compared with women with ID, men with ID reported better access to HIV information through parents, siblings, friends, and teachers [107]. People with ID face significant barriers to knowledge about HIV, including misconceptions about sexual activity, misinformation, negative community beliefs and attitudes among service providers, and literacy challenges [108]. Facilitators identified comprised participatory responses to stigma, employing messages relevant to persons with ID and increasing this group’s active and visible participation in intervention activities [108].

On the other hand, people with ID had significantly lower odds of an STI diagnosis [109]. HIV testing prevalence was similarly lower among people with ID, with non-significant gender differences [106].

### 3.5. Sexuality and Sex Education

This theme is made up of 28 articles. The vast majority (*n* = 22; 78.5%) were published in the last decade. Twenty studies included participants with ID (who represented 100% of the sample in 10 of the articles). In the others, people with ID were part of a larger sample that also comprised people without ID, allowing comparison between both groups. Of the eight articles whose samples were not composed of people with ID, the participants tended to be mothers, health professionals, or teachers. Although most studies included participants of both genders, more participants were female. Nine studies specified the level of ID, with mild ID being the most frequent. One study included participants with severe ID, but none included participants with profound ID. 

The following subthemes were identified: (a) knowledge; (b) barriers and facilitators; (c) sexual intercourse experiences; and (d) interventions.

#### 3.5.1. Knowledge

Ten articles were categorized into this subtheme [21,56,62,110,111,112,113,114,115,116]. Four studies involved people with ID (representing 100% of the sample in two of them), while the sample was composed of health workers or staff in two studies.

The research highlighted personal autonomy and agency as fundamental for the development of gender and sexual identities [62]. Accessible information and education about sexuality and relationships were also needed for young people with ID [111]. Although all participants had received sex education once or twice during their life [50], the results showed an extremely low level of knowledge about sexuality and sexual intercourse, with especially limited knowledge about pregnancy, contraception, and STIs [110,113,114,115]. The topics mentioned do not cover the entire area of sexuality [56]. 

An education program for adolescents with disabilities must cover topics such as body parts as well as physical and physiological changes. In this regard, doctors reported that available sexual knowledge assessment tools for people with ID were essentially all similar in structure and content. However, with publication dates ranging between 1994 and 2006, all need to be brought up to date as they tend to miss essential sexuality topics [116]. 

#### 3.5.2. Barriers and Facilitators

This subtheme is made up of nine articles [118,119,120,121,122,123,124]. Six studies included men and women with ID but did not specify the degree of ID. Four studies included other participants in the sample, mainly teachers (*n* = 3). The barriers and facilitators identified came from people with ID and the educational staff working with them.

The studies highlighted the following as barriers [119,123,124]: the systemic restriction of access to information about sexual and reproductive health and rights (SRHR); the lack of sex education training; unsupportive home environments, combined with low socioeconomic status and cultural upbringing; overprotective families; perceived conflict between informants’ values and religious ideologies; not enough training for specialists; and the age of educators (younger educators perceived themselves to be more flexible and able to challenge thinking, social beliefs, and norms as they saw themselves as more accepting of change because they were “open and broad-minded”). 

The studies identified the following as facilitators [117]: consideration of individual differences; cooperation with parents; choosing appropriate environments; sanitation rules; and receiving information from a same-sex specialist.

The school environment was a crucial contextual factor that could function as an enabler or a barrier [119]. Participants highlighted three characteristics in particular: (a) organizational features, including leadership style and management; (b) school facilities and infrastructure; and (c) the teacher–student relationship.

#### 3.5.3. Sexual Intercourse Experiences

Four articles fell into this subtheme. Three [126,127,128] were quantitative and had been carried out in the USA. The other paper [125] was qualitative and had been conducted in the UK. The four articles included males and females with ID, mostly adolescents (12–17 years). In all studies, the sample was also composed of people without ID or with other disabilities. 

Those with ID were more likely to report a very early sexual debut (12–14 years) and to talk about birth control, but they were less likely to use condoms when they used contraceptive methods [128]. Girls with severe ID who did not use contraception at their first experience of sexual intercourse were also much more likely to want a pregnancy [127].

#### 3.5.4. Interventions

Five studies were included [129,130,131,132,133]. Spain stands out as the country with the highest number of interventions (*n* = 2.). Most of the studies were qualitative (*n* = 3) and two were quantitative. All were carried out with people with ID (the participants were male and female in four of them, while one targeted adolescent girls exclusively). All interventions addressed mild-to-moderate ID.

In an intervention with a Real-Care-Baby (RCB) simulator [133], students described the educational material in favorable terms and found that most of what they learned was new information. They generally voiced appropriate and realistic expectations about parenthood and stated that the combination of theoretical knowledge and practical experiences helped them to grasp the notion of parenting. 

In another intervention with people with ID [129], groups were set up to discuss issues related to sex education and relationships. Participants’ general experience was positive. They preferred the groups to be facilitated by male and female administrators together and also to have the sex education sessions before the relationship sessions (which helped them better explore the sessions through a knowledge base of sexuality topics and terminology).

Another empirical study [132] carried out with people with ID offered evidence demonstrating the usefulness of a brief intervention program to improve the knowledge and attitudes toward consensual and responsible sexual relationships.

The fourth intervention with people with ID [130] created feminist dialogue groups, which were found to have a positive impact on the lives of adolescent girls with ID, specifically by promoting preventive interactions that would protect them from gender-based violence in their relationships.

The remaining intervention [131] targeted mothers of girls with ID. The training was more effective when conducted in face-to-face participatory groups rather than reading a manual alone, despite a manual being created specifically for this purpose. The results showed that the mean scores of mothers’ awareness, attitude, and self-efficacy in caring for the sexual health of their daughters with ID were significantly higher after the intervention. The results also showed that one month after the intervention, the mean score was higher in the “group training” participants.

### 3.6. Pregnancy

This theme was made up of 33 articles. The vast majority were quantitative and based on medical records or large health survey databases. Only three were qualitative. Only two studies were published before 2006, and the other 31 were published from 2012 onwards. Participants in all studies were women with ID, with an average age ranging between 20 and 40 years, and only three studies specified the degree of ID (mild in all of them, moderate in two, and severe in one). In four studies, women with ID represented 100% of the sample. In the remaining cases, their results were compared either with other women without ID (*n* = 25) or with other disabilities (*n* = 12). 

Four subthemes were identified: (a) the profile of women with ID who become pregnant; (b) their prenatal, pregnancy, and postnatal health; (c) birth outcomes; and (d) barriers and facilitators encountered during pregnancy.

#### 3.6.1. Profile of Pregnant Women with ID

Women with ID who became pregnant tended to have the following characteristics: they were under 20 years old; they were living in low-income neighborhoods and rural areas; their ethnicity was non-Hispanic Black or Hispanic; they had public health insurance; and they were not married [134,135,140,143,144,145,150,155,156,158]. They were also at increased risk for rapid repeat pregnancy within a year of a live birth [144].

#### 3.6.2. Health Conditions before, during, and after Pregnancy

Women with ID generally received inadequate prenatal care [152], sometimes because they had difficulties identifying the signs of pregnancy [164]. They also tended to have modifiable risk factors, such as alcohol use, tobacco use, and obesity [149,150,152]. They had more complications during the pregnancy (gestational diabetes, preeclampsia, eclampsia, venous thromboembolism, and severe obstetric morbidities, such as placental abruption) [135,140,143,145,146,148,150,153,155,156,158,159,160] but were less likely to receive cervical cancer screening [134]. Preexisting health conditions and maternal complications explained some of the elevated occurrences of labor inductions and cesarean sections [142,162,163]. Findings also identified a pattern of unlabored cesarean deliveries that did not appear to be medically indicated [138]. This finding raises the possibility that ID may be treated as an indication for C-sections in many cases. In this sense, the research studies that have analyzed this issue point out that there is a clear need for healthcare professionals (especially gynecologists and obstetricians) to carefully weigh up the benefits and risks of such surgery in order to recommend the most appropriate course of action to a patient with ID. This assessment and the subsequent recommendation have been highlighted among the most important aspects of care for patients and mothers-to-be with ID [138,142,162].

#### 3.6.3. Birth Outcomes

Of the live births recorded, the babies of women with ID were more likely to be born preterm, to be small for their gestational age, to experience neonatal morbidity, to die in the first month of life, or to be more frequently admitted to a neonatal intensive care unit. Stillbirth was almost four times more prevalent, and perinatal death was more than four times more common [151].

Women with ID had a particularly high risk for custody loss: 1 in 20 newborns of women with ID were discharged to child protective services immediately after the birth hospitalization. Furthermore, children of mothers with ID had a three times higher risk of being exposed to injuries, violence, and child abuse compared with children of mothers without disabilities [166].

#### 3.6.4. Barriers and Facilitators

The barriers most frequently cited by the women participants with ID included the following: negative attitudes and judgments of healthcare staff about their pregnancy [139]; communication barriers, especially because of biomedical jargon [162]; feeling pressure to demonstrate their ability to be good mothers [139,154,157,162,165]; and loss of control [139]. In general, women with ID who were also members of ethnic minority groups faced greater barriers to healthcare access and received lower quality services [134].

Facilitators included the following: finding support and positive attitudes among healthcare staff; having a good support network (both family and professional); and empowering knowledge (content, amount, and accessibility) [154].

### 3.7. Experiencing Parenthood

This last theme was made up of four studies [167,168,169,170], all of which were published after 2019. All were qualitative, and the results were obtained through in-depth or semi-structured interviews. Two focused on the experience of mothers, one on the experience of fathers, and one on the experience of daughters whose mothers had ID.

Mothers with ID participating in the studies included in this theme [167,168] were threatened by domestic violence, and neither of the two articles focused on positive experiences. For many of the participants, domestic violence also marked the beginning of their motherhood (e.g., violent conceptions). The experience of violence posed two major threats for women, namely, the possibility of losing custody of their children and the barriers they faced because of triple discrimination (being a woman, having ID, and being a victim of violence).

Fathers with ID [169] mentioned the challenges of parenthood, both logistically (e.g., paying bills) and emotionally (e.g., dealing with tantrums), which sometimes made them feel insecure. Much of the support they received was focused on non-parenting aspects and was more directed at mothers, making them feel a sense of exclusion.

Daughters of mothers with ID [170] highlighted that their childhood had been filled with neglect, deprivation, anxiety, responsibility overload, and a lack of stability and support. The daughters emphasized how their childhood experiences of having a mother with ID had influenced their life decisions and their health into adulthood. In this study, the researchers pointed out that these participants, then in their 40s and 50s, had grown up in a social context and at a time in history when child protection social services were not what they are today. In other words, the consequences of having a mother with ID can be closely linked to the lack of support due to the functioning of the system 30 years ago.

## 4. Discussion

By synthesizing current qualitative and quantitative evidence related to Articles 23 and 25 of the CRPD, this review set out to ascertain whether the right to home and family and the right to sexual and reproductive health of people with ID is respected. To answer this question, the review summarizes what the scientific literature says about attitudes toward the sexuality of people with ID and also what it says about barriers and facilitators that people with ID encounter in exercising their right to home and family and their sexual and reproductive rights.

The 151 articles reviewed show that interest in the rights covered by Articles 23 and 25 of the CRPD has been growing since 2006, the year the Convention was adopted. These topics attracted limited research attention prior to the CRPD. In terms of the topics addressed before 2006, there was a marked focus on SRH and on contraceptive choices in particular. The topics broadened considerably after 2006. Although there was still a clear focus on SRH, the research became more centered on the barriers and facilitators faced by people with ID, and this aspect was particularly relevant for papers published after 2010.

Despite the progress that has been made, the results of this review show that there are still many barriers and much work to be done in order to guarantee the fulfillment of these rights for people with ID. Gómez et al. [10,16] have highlighted the many situations of rights abuse that still occur today, from the perspective of people with ID themselves and from that of the professionals working with them and their families. For women with ID, the situation is further compounded. This group faces greater discrimination on the basis of their disability but also their gender. With their femininity considered as “defective” and with families and people in their close network regarding them as “eternal girls” [173], access to their rights is further restricted.

Like any other person, people with ID are sexual beings. Although the common rights stipulated by the CRPD are increasingly respected [174], there is still frustration, uncertainty, and stigma. The results presented in this review suggest that community attitudes are skeptical about people with ID becoming parents. As some research has pointed out [175,176], those with power, authority, and influence over women with ID usually consider it their responsibility to try to prevent them from having children. Other factors influencing attitudes toward the sexuality of people with ID include age (with older respondents holding fewer liberal attitudes), knowledge and education, cultural norms, and religious beliefs. 

One crucial aspect that has emerged from this review is that people with ID want to be emotionally attached to others. Affectionate relationships are a symbol of a good life and give a sense of normality [177]. There are, however, many barriers that make it difficult for this population not only to enter into a loving relationship with another person but also to maintain it. These findings are in line with the concerns raised by other researchers about people with ID being stigmatized and isolated [178]. When it comes to supporting needs, this review finds that relationship support tends to focus all the attention on risk and vulnerability to abuse, especially for women [179]. There is a dilemma that exists among staff, who want people to have relationships but fear for their safety. In addition, staff feel like they are “walking the tightrope” when they have little or no training and no knowledge of organizational policy. 

One observation in relation to marriage is the value it has for many cultures, which increases the risk of people with ID being forced into a marriage, sometimes because parents of people with ID believe that marriage may cure their offspring’s disability [180]. Most studies have reported that abuse in people with ID (mostly girls and women) is more likely to come from a known person [181], and forced marriages seem to provide the perfect setting for these acts to take place or to be hidden and even legitimized. If the barriers to intimate relationships can perpetuate violence against and the abuse and forced marriages of people with ID, it is then crucial that the removal of these barriers is prioritized and included within policies and strategic plans.

Sexual and reproductive health (SRH) is a vital but often neglected and stigmatized aspect of healthcare for people with ID [182]. Studies included in this review have documented barriers to healthcare access for people with ID across the life spectrum. Other empirical investigations [183,184,185] have discussed similar barriers to those identified in this review, highlighting three major categories: (a) individual characteristics, such as gender, socioeconomic factors, sociocultural norms and beliefs, and severity or degree of ID; (b) nonmedical systemic factors, such as architectural designs or infrastructure; and (c) providers’ attitudes, perspectives, and appreciation of the needs of people with ID. Lack of skills and awareness training for health professionals, communication barriers, and stigmatizing attitudes represent the main barriers to SRH access. 

One of the overarching findings of this review is that women with ID encounter barriers in their access to SRH. This is consistent with previous studies [186], which found that male participants were less likely than female participants to experience unmet support demand. Many women with ID are capable of playing an active and central role in healthcare decision-making but often feel excluded from decisions and believe that health professionals underestimate their abilities. This is especially true when it comes to decisions about contraception or family planning [187]. The phrase “informed compliance rather than informed choice” aptly describes the situation of many women with ID when decisions are being made about their reproductive health [176]. The results from our review coincide with findings from similar research, showing that women with ID tend to take contraception when they are not sexually active to prevent pregnancy, manage menstruation, and, disturbingly, protect against abuse [188,189]. However, it is important to reiterate that contraception or sterilization does not prevent sexual abuse [183]. Moreover, most adolescents with ID have unique medical, technical, and social needs, particularly in the area of menstrual hygiene [190,191]. There is a lack of social support and information about how to care for another person’s menstrual cycle. Menstrual care is viewed as a private issue, and this results in carers feeling overwhelmed and isolated, as other studies have also highlighted [192,193,194,195,196]. 

Additionally, the social perception of people with ID as *asexual* leads to them being excluded from sex education. People with ID sometimes internalize the message that any sexual expression is unacceptable based on people’s attitudes and behaviors toward them (including overprotective attitudes and behaviors) [197]. Furthermore, caregivers are usually unaware of—or disapprove of—the person with ID engaging in sexual activity, leading them to believe that talking about certain topics may awaken behaviors they want to keep dormant [198]. Research has shown that, compared with people without disabilities, people with ID have limited knowledge of sexuality-related topics. Sex education is essential and must be provided, considering individual differences and in cooperation with parents, since it is clear that adults with ID engage in sexual relationships and have sexual intercourse [199,200].

When it comes to pregnancy, one of the conclusions to be drawn from most of the studies reviewed is that women with ID are considered to be a risk group. They are less likely to receive prenatal care during the first trimester, which usually leads to C-section deliveries. In addition, women with ID tend to be younger, less educated, and on lower incomes compared with pregnant women without ID [201,202]. Communication barriers, deficits in health information, negative attitudes toward pregnancy in women with ID, and a lack of knowledge and awareness among healthcare professionals were identified as key issues in the scientific literature [203,204,205,206]. Midwives reported having insufficient training or time during appointments to provide adequate support to women with ID [207]. Furthermore, many participants in the reviewed studies felt that they could not take motherhood for granted, having to prove to the authorities and to some family members that they were able to be suitable parents.

Despite the many positive aspects of parenthood, such as increased satisfaction and self-esteem, and the fundamental role it plays in developing a positive identity, a sense of femininity, and motivation and meaning in life, the reviewed literature does not devote much attention to parents with disabilities and this kind of experience. Parenting is a social construct, and therefore, there is an ideal parenting model. Disability is so closely associated with dependency and social isolation that people find it difficult to imagine a person with ID at the center of family life in the role of the primary caregiver [208], an argument that may be underlying the fact that the included studies only highlight the difficulties and risks of parenthood in people with ID. Although not all maternity experiences are connected to domestic violence, it is noteworthy that two of the four studies included in this topic take this factor into account. Domestic violence is a determinant factor in the decision to remove children from families headed by parents with ID, and indeed the biggest fear of parents with ID is losing custody of their child to statutory care [209,210]. In the case of child removal, parents with ID reported feeling unfairly treated, powerless, and unsupported [207,209]. From the other angle, when we look at the perspectives of children whose parents have ID, we can identify a variety of obstacles that might prevent the children from receiving proper care, including limited awareness of the child’s needs, the child having to take care of family members, and, above all, a lack of support from their families and institutions. When good support is provided early, parents with ID care successfully for their children [211].

This systematic review has some limitations. First, relevant studies may have been missed from the review despite the inclusion of general and specific descriptors. During the search procedure, to determine which articles met the inclusion criteria, the researchers considered a large number of search terms, but these specific terms may have biased the selection of articles and important studies could have been overlooked. It is, therefore, important to notice the potential presence of both selection and reporting biases. Second, given that participants with severe and profound ID are under-represented in the studies, the conclusions drawn cannot be extrapolated equally to them. Similarly, it would be useful to study gender differences in greater depth, increasing the number of studies with a similar number of male and female participants. On topics such as parenthood, for instance, there is scant empirical literature that collects narratives from the point of view of fathers, so it would also be important to consider this aspect in future research. Finally, more robust research methodologies could be used since the tendency in most studies is to include very small convenience samples of people with ID, and in many stances, data were collected for a different initial purpose than the study they subsequently appeared in, leaving relevant information uncollected. Additionally, more exhaustive filtering of the date study publication dates would enable a more in-depth analysis of the issues dealt with before and after the CRPD. Finally, the large number of studies found, although this is a noteworthy aspect given the extensive information reviewed, can also be considered a limitation. This issue has made it necessary to organize the results into categories. However, more specific systematic reviews may also be necessary to address each topic detected, or we should even consider carrying out meta-analyses beyond a systematic review.

Despite these limitations, this review has strengths worth mentioning. First, this is a reproducible and reliable review following the most recent PRISMA guidelines, thus reducing variability and uncertainty and making it possible to manage scientific information to obtain conclusions on this relevant topic. The high percentage of interrater agreements should also be pointed out. Second, this review deals with two hugely important CRPD rights that span a wide range of subthemes covered in a large number of studies. These two strengths have enabled the authors to present the current scientific knowledge on this topic and thus address the objectives and questions that were initially raised. The conclusions contribute to scientific progress by responding to the gaps and needs detected and by proposing new directions and improvements for future research.

## 5. Conclusions

Effective implementation and monitoring of the CRPD for people with ID is a clear and imperative priority in most countries [10]. For this to become a reality, a holistic understanding of sexual and reproductive health and rights is required, recognizing that “good” sexual and reproductive health is not simply the state of being free from disease or injury but rather “a state of physical, emotional, mental and social wellbeing concerning all aspects of sexuality and reproduction” [176]. Consequently, the achievement of good health outcomes is indisputably based on the respect and promotion of human rights, including the right of all persons to be respected for their bodily integrity, privacy, and personal autonomy; the right to freely define their sexuality, including sexual orientation and gender identity and expression; the right to decide if and when they are sexually active, to choose their sexual partners, and to have safe and pleasurable sexual experiences; and the right to make decisions about related matters, such as sexual relations, marriage, civil union, divorce, contraception, sterilization, and termination of pregnancy, with access throughout their lives to the information, resources, services, and support necessary to achieve all of the above, free from discrimination, coercion, exploitation, and violence [176,212].

To contribute to scientific progress by addressing the gaps and needs detected in this review, the quality of life (QOL) paradigm, in particular, the Quality of Life Supports Model [213], is the most appropriate conceptual framework for translating concepts as abstract as self-determination, equity, accessibility, or inclusion into practice and policy. It is also the ideal theoretical space to promote the effective fulfillment of the principles and rights contained in the CRPD, facilitating the simultaneous action of both individuals and the environments in which they live [4,10,214]. Gómez et al. [215] followed this premise and theoretical framework in order to develop useful indicators to operationalize, measure, and implement the Articles of the Convention on the Rights of Persons with Disabilities (CRPD). Verdugo et al.’s [214] and Lombardi et al.’s [8] papers gave clues to achieving this goal. Verdugo et al. [214] presented an initial proposal to organize the 26 CRPD Articles by QOL domain, with Articles 23 and 25 linked to the interpersonal relationships and physical wellbeing domains. Likewise, Lombardi et al. [8] described the international consensus on the relationship between the core quality of life indicators and the articles of the CRPD, and Gómez et al. [9,10] reported the Spanish consensus indicators for its assessment. Following these statements, Table 4 summarizes the relationship between these Articles of the CRPD with the QOL domains and indicators, including the main themes found throughout this review.

## Figures and Tables

**Table 1:** Boolean terms used in the six searches carried out in the databases.

Search 1	intellectual disabilit* OR intellectual impairment OR mental retardation OR developmental disabilit* OR learning disabilit* OR down syndrome* OR fragile x* AND marriage OR marital OR couple OR spouse
Search 2	intellectual disabilit* OR intellectual impairment OR mental retardation OR developmental disabilit* OR learning disabilit* OR down syndrome* OR fragile x* AND “family planning” OR “contraceptives methods” OR contraception OR “birth control”
Search 3	intellectual disabilit* OR intellectual impairment OR mental retardation OR developmental disabilit* OR learning disabilit* OR down syndrome* OR fragile x* AND women OR woman AND maternity OR motherhood OR mother OR maternal OR mothering OR pregnancy OR pregnant OR parenthood OR parents OR parenting
Search 4	intellectual disabilit* OR intellectual impairment OR mental retardation OR developmental disabilit* OR learning disabilit* OR down syndrome* OR fragile x* AND “romantic relationship” OR “close relationship” OR “intimate relationship” OR boyfriend OR girlfriend
Search 5	intellectual disabilit* OR intellectual impairment OR mental retardation OR developmental disabilit* OR learning disabilit* OR down syndrome* OR fragile x* AND woman OR women AND “menstrual education” OR “menstrual support” OR “reproductive health”
Search 6	intellectual disabilit* OR intellectual impairment OR mental retardation OR developmental disabilit* OR learning disabilit* OR down syndrome* OR fragile x* AND sterili* OR “forced sterili*”

Note: * symbol includes singulars, plurals and other combinations to broaden the scope of the search (e.g.: sterili*: sterilized, sterilization).

**Table 2:** Criteria agreed upon by the authors for the inclusion of studies in the review.

Study design	Empirical studies: Any qualitative, quantitative, or mixed-method observational study containing attitudes, interventions, and primary or secondary data analysis published in peer-reviewed scientific journals.
Aim of the study	Studies about issues relating to Article 23 (home and family) or Article 25 (health, specifically sexual and reproductive health) of the CRPD: This includes studies covering such diverse topics as affective, loving, or intimate relationships; marriage or living as a couple; maternity, paternity, founding a family, and bearing children; family planning, contraception, and sterilization; sexual and reproductive life; relationships and sex education.
Participants and sample	Two types of studies according to participants: (a) Studies whose participants were people with ID (including those with specific syndromes such as Down Syndrome or Fragile X) and studies that clearly described a subgroup with ID; and(b) Studies whose participants were family members, caregivers, or health providers of people with ID.
Language	Studies written in English or Spanish.

**Table 3:** References included in the review organized by themes.

Theme (*n* Studies)	References Included in This Review (Numbers Used in the Reference List)
Attitudes (*n* = 17)	[20,21,22,23,24,25,26,27,28,29,30,31,32,33,34,35,36]
Intimate Relationships (*n* = 30)	[22,37,38,39,40,41,42,43,44,45,46,47,48,49,50,51,52,53,54,55,56,57,58,59,60,61,62,63,64,65]
Sexual and Reproductive Health (SRH) (*n* = 47)	[21,35,62,66,67,68,69,70,71,72,73,74,75,76,77,78,79,80,81,82,83,84,85,86,87,88,89,90,91,92,93,94,95,96,97,98,99,100,101,102,103,104,105,106,107,108,109]
Sexuality and Sex Education (*n* = 28)	[21,33,56,62,110,111,112,113,114,115,116,117,118,119,120,121,122,123,124,125,126,127,128,129,130,131,132,133]
Pregnancy (*n* = 33)	[134,135,136,137,138,139,140,141,142,143,144,145,146,147,148,149,150,151,152,153,154,155,156,157,158,159,160,161,162,163,164,165,166]
Experiencing Parenthood (*n* = 4)	[167,168,169,170]

**Table 4:** Relationship between Articles 23 and 25, QOL domains, and the review’s main themes.

CRPD Articles	QOL Domain Based on Verdugo et al. [214]	QOL Indicators Based on Gómez et al. [9,10] and Lombardi et al. [8]	Topics and Subtopics
23 (Respect for home and the family)	Interpersonal relationships	Right to set up their own family Right to be a parent Dating and intimacy with persons of choice	Attitudes Intimate Relationships Pregnancy Sexuality and Sex Education Experiencing Parenthood
25 (Health)	Physical wellbeing	Physical status Nutritional status Chronic conditions	*Attitudes Toward Sterilization* Sexuality and Sex Education Sexual and Reproductive Health (SRH) Pregnancy

Note: *italics* refers to the specific subtopics included in the Attitudes topic linked to Article 25 and the physical wellbeing domain.

## Data Availability

Not applicable.

## Supplementary Materials

The following supporting information can be downloaded at: https://www.mdpi.com/article/10.3390/ijerph20021587/s1, Table S1: Theme One. Attitudes (*n* = 17); Table S2: Theme Two. Intimate Relationships (*n* = 30); Table S3: Theme Three. Sexual and Reproductive Health (*n* = 47); Table S4: Theme Four: Sexuality and Sex Education (*n* = 28); Table S5: Theme Five: Pregnancy (*n* = 32); Table S6: Theme Six: Experiencing Parenthood (*n* = 4).
