# Supplementary material for: The Right to Sexuality, Reproductive Health, and Found a Family for People with Intellectual Disability: A Systematic Review
Source: Int J Environ Res Public Health. 2023 Jan 15;20(2):1587. doi: 10.3390/ijerph20021587 (PMC9864803; doi:10.3390/ijerph20021587)
Supplement: Supplementary file 1 [file ijerph-20-01587-s001.zip › ijerph-2065134-supplementary.pdf]

Table S1. THEME ONE. ATTITUDES (n=17)

Study citation. Year. Country	Subtheme	Aim of the study	Type of study and design. Instrument and measures.	Includes participants with ID	Sample*	Main findings
**Bornman et al. (2016) South Africa [20]**	T. Sexuality	Develop four social stories that could be used as part of a sexuality and relationship training program for WWID.	Qualitative<br>Thematic analysis<br><br>Multi-method study<br>Focus groups	Yes	Women with ID<br><br>female<br><br>23-55<br><br>n=10 (100%)	The directors at adult facilities regarded sexuality training as inappropriate. They expressed a fear that training would result in an increase in sexuality behaviour in women with ID a behaviour they were actively trying to discourage and suppress. Some claimed that women with ID were "asexual". These findings showcase the misconceptions and myths related to the sexuality of women with ID.
**Christian et al. (2001) USA [21]**	T. Sexuality	Determine the attitudes and knowledge of support staff at an agency serving individuals with IDD.	Quantitative<br><br>Survey	No	Staff<br><br>both; mostly female<br><br>+18<br><br>n=43 (100%)	Most respondents agreed that women with ID have the same sexual desires as women without disabilities. Almost all agreed that sexuality is an important part of who we are and that women with disabilities should have the freedom and opportunity to express their sexuality. Most staff agreed that service providers were responsible for addressing the sexual wants and desires and supporting their sexual expression, but less than half felt that service providers recognized women with disabilities as sexual beings.
**Deffew et al. (2022) Ireland [22]**	T. Sexuality	Investigate staff views and attitudes considering the impact of variables on these attitudes.	Quantitative<br><br>ASQ-ID<br>Questionnaire	No	Staff<br><br>both; mostly female<br><br>18-65+<br><br>n=86 (100%)	Staff recognised that love is a human desire and we need to give the people we support an opportunity. However, they reflected how the lack of open discussion had repressed people with ID expression of sexuality. They detailed how religious teachings were very much part of the founding culture, and these viewpoints still existed in services today. Staff reported that issues around people with ID sexuality were often about negative experiences. This reactionary method is related to their own Sexual Education.
**Gilmore & Chambers. (2010) Australia [23]**	T. Sexuality<br>T. Parenting<br>T. Sexual freedom	The present study examined attitudes in a sample of disability support staff.	Quantitative<br><br>ASQ-ID<br>ASQ-GP<br>Questionnaire	No	Disability support staff<br>Leisure and service industries<br><br>both male and female<br><br>20-70+<br><br>n=219 (100%)	Attitudes towards sexuality<br>Both groups of participants demonstrated generally positive attitudes, however it is still less accepted compared to typically developing adults. Gender had a significant influence on the attitudes.<br>Attitudes towards parenting<br>Support staff were less positive about parenthood than other aspects of sexuality.<br>Attitudes towards sexual freedom<br>Support staff saw it acceptable for typically developing (TD) individuals than for individuals with an ID. Respondents saw less sexual freedom as desirable only for females with an ID. Both groups of respondents see men with an ID as having less self-control over their sexual behaviour.

Study citation. Year. Country	Subtheme	Aim of the study	Type of study and design. Instrument and measures.	Includes participants with ID	Sample*	Main findings
**Gilmore & Malcolm. (2014) Australia [24]**	T. Sexual freedom<br>T. Sexuality<br>T. Sterilization<br>T. Parenting	Part 1. Investigate doctors' attitudes towards sexuality.<br>Part 2. deepen understanding of the attitudes towards sterilisation.	Quantitative<br><br>ASQ-ID<br>ASQ-GP<br>Modified version of the questionnaire (sterilisation)	No	Doctors<br><br>both male and female<br><br>20-70+<br><br>n=203 (100%)	<u>Attitudes towards the sexual freedom</u><br>Participants indicated less support for the sexual freedom of adults (both men and women) with ID.<br><u>Attitudes towards sexuality</u><br>Views were generally positive and similar towards male and female sexuality.<br><u>Attitudes towards parenting</u><br>Views were found to be significantly less positive about parenting than about other aspects of sexuality. More negative towards women.<br><u>Attitudes towards sterilization</u><br>Pattern of responses found. Sterilization is a desirable practice for both men and women with ID. Older doctors reported more support for sterilization.
**Jones et al. (2010) Australia [25]**	T. Sexuality<br>T. Parenting	Rate attitudes amongst different student groups.	Quantitative<br><br>Intervention and non-intervention group<br>Pre and post (Cuskelly) Questionnaire	No	-<br><br>-<br><br>-<br><br>-	Results indicate that attitudes trend more positively with a small minority reporting a negative attitude towards the sexuality and parenting of people with ID. Students related to disability fields are more positive than others. Younger students hold more positive attitudes towards women with ID becoming parents.
**McCarthy et al. (2021). UK [26]**	T. Marriage	Better understand the risk factors which may contribute to forced marriage amongst people with ID.	Qualitative<br>Thematic analysis (Braun and Clarke)<br><br>Focus group<br>Semi-structured interview	No	-<br><br>-<br><br>-<br><br>-	There was unanimous agreement amongst participants that marriage was a central tenet of their faith and culture. Notably, some participants felt that this cultural or community pressure directly led to a lack of openness about marriages, including forced marriages, involving people with ID. However, most participants showed resistance to marriage, which was not only associated with perceived characteristics and abilities of individual with ID, but also with participants' wider fears about the potential consequences of marriage.
**Morales et al. (2010). Mexico – France [27]**	T. Sexuality	Compare the attitudes of laypeople from two different national communities: Mexican and French.	Quantitative<br><br>Application of Anderson's Functional Theory of Cognition (Esterle et al.) question and a response scale	No	General population<br><br>both; mostly female<br><br>22-75<br><br>n=120 (100%)	<u>Attitudes towards sexuality</u><br>Comparing Mexican's and French's adults toward the expression of sexuality by persons with learning disabilities, Mexican participants seem to be more open. Only three factors were found to have a significant effect: Contraception, Autonomy, and Partner's age. In France, sexuality without contraception is considered to be completely unacceptable, irrespective of the other circumstances.

Study citation. Year. Country	Subtheme	Aim of the study	Type of study and design. Instrument and measures.	Includes participants with ID	Sample*	Main findings
**Morales et al. (2011). Mexico [28]**	T. Sexuality	Comparing people's attitudes (family caregivers, professional care, special education teachers, and other personals in charge of PLD.	Qualitative<br>Cluster analysis<br><br>64 vignettes Esterle et al.<br>Focus group	No	Proxies<br><br>-<br><br>19-63<br><br>n=75 ("-" %)	Findings suggest it may be erroneous and risky to consider the parents of PLD as a homogeneous group regarding attitudes to sexuality. The more common identified attitude was Mainly Unacceptable. Factors influencing acceptability were found. Contraception was by far the major determinant of acceptability.
**Muswera & Kasiram. (2019). South Africa [29]**	T. Sexuality<br>T. Parenting	Understand the sexuality of persons with disability living in residential care facilities.	Qualitative<br>Thematic content analysis<br><br>Individual interviews (service users)<br>Focus groups discussions (service providers)	Yes	People with ID<br><br>-<br><br>20-55<br><br>n=4 (66%)	<u>Attitudes towards sexuality</u><br>There are many misconceptions related to the sexuality of persons with disability.<br><u>Attitudes towards parenting</u><br>Considered people with ID not being candidates for marriage and parenthood. No change for them to be in a healthy marriage or have any children.
**Pajot et al. (2015). France [30]**	T. Parenting	Map people's views regarding childbearing among PLD.	Quantitative<br>Cluster<br><br>Questionnaire (60 realistic stories and a 10-point response scale)	No	General population<br><br>both; mostly male<br><br>18-84<br><br>n=304 (100%)	<u>Attitudes towards parenting</u><br>A group of participants, elderly and very religious, considered child bearing was not reasonable. Other group, aged less than 26 and self-identified leftists or centrists, considered that parenting was quite reasonable for PLD even in severe LD cases. The majority group considered parenting was acceptable when the LD was light and the relationship between partners was an enduring one.
**Parchomiuk. (2012). Poland [31]**	T. Sexuality	Determine which emotional and evaluative attitudes towards various aspects of sexuality are presented by professionals who support people with ID.	Quantitative<br><br>Semantic differentials questionnaire	No	-<br><br>-<br><br>-<br><br>-	It was found that attitudes of specialists towards sexuality of people with disabilities differ substantially. The respondents find it more difficult to accept certain phenomena associated with the physical and psychosocial dimensions of sexuality of individuals with ID. With a lower acceptance, aspects of partnerships and parenthood were found. Special educators turned out to be less supportive in most of the evaluated dimensions.
**Parchomiuk. (2013). Poland [32]**	T. Sexuality<br>T. Sterilisation	Explore students' attitudes towards the sexuality of persons with ID, especially regarding the emotional-evaluative and cognitive aspect.	Quantitative<br><br>''The model of an intellectual disability'',<br>"The essence of sexuality",<br>Questionnaire	No	University students<br><br>both; mostly female<br><br>22 average<br><br>n=181 (100%)	<u>Attitudes towards sexuality</u><br>Sex education and emotional-evaluative attitude obtained the most positive results. Satisfaction of sexual needs of people with ID, both in the form of sexual intercourse and masturbation, was not accepted by the participants.<br><u>Attitudes towards sterilisation</u><br>Perceiving ID as a biological, irrevocable phenomenon is related to the low valuation of the sexuality of people with ID, and leads to accepting sterilization as a method of blocking fertility permanently.

Study citation. Year. Country	Subtheme	Aim of the study	Type of study and design. Instrument and measures.	Includes participants with ID	Sample*	Main findings
**Pownall et al. (2012) UK [33]**	T. Sexuality	Compare attitudes and behaviours of mothers of young people with and without ID.	Mixed method<br><br>In-depth interview Questionnaire	Yes (both)	Young people with mild ID<br><br>-<br><br>16-24<br><br>n=30 (50%)	The mothers with offspring with ID were more cautious in their attitudes toward sexuality. The most different item was their perception of their child's vulnerability to sexual abuse, agreeing they are more vulnerable than other young people. Other items with a large discrepancy concerned the young people's ability to make responsible decisions about contraception and sexual relationships, readiness to learn about sexual matters, and desire for intimate relationships. Mothers were more negative towards their child engaging in sexual intercourse, getting married and having children of their own.
**Taghizadeh et al. (2020) Iran [34]**	T. Sexuality<br>T. Marriage<br>T. Parenting	Discover the opinions and beliefs of caregivers about sexual needs and marriage of WWID.	Qualitative Conventional content analysis method<br><br>In-depth individual semi-structured interviews	No	Proxies<br><br>-<br><br>28-62<br><br>n=21 (100%)	<u>Attitudes towards sexuality</u><br>All participants believed that sexual desire is natural and undeniable. They also believed that sexual need is not related to the degree of mental capacity. However, fulfilling these sexual needs is limited due to many obstacles. Some caregivers consider the presence of cultural taboos as an obstacle for women with ID.<br><u>Attitudes towards parenting</u><br>Health professionals pointed out that and in the case of childbearing, they should be supervised by doctors, psychologists and family consultants but it is better not to have children. And, when ID is genetically inherited, or there is the possibility of neglect and inability to care for the child, it should be prevented.<br><u>Attitudes towards marriage</u><br>Most caregivers did not agree with their marriage as they do not have the ability to maintain marital relationships imposing a double burden on the family. In contrast, most mothers of children with ID wanted their children to marry, and believe that marriage is a way to achieve tranquillity for their children and a way to guarantee their future. Health professionals believed that women with ID, as with other people have the right to marry and to start a family, but the conditions for marriage and family formation must be provided in advance.
**Wickström et al. (2020) Sweden [35]**	T. Sexuality<br>T. Parenting	Gain a deeper understanding of staff's experiences and perceptions regarding sexual and reproductive health and rights (SRHR) related to individuals with ID.	Qualitative<br><br>Focus groups	No	Staff that worked with people with mild-to-moderate ID<br><br>both male and female<br><br>18-65<br><br>n=20 (100%)	<u>Attitudes towards sexuality</u><br>The staff noticed a positive progress in society. They agreed that sexuality and sexual identity are human rights for everybody. They pointed remaining challenges such as frustration, uncertainty and stigma. Some had experienced that sexuality of individuals with ID evokes negative emotions in society.<br><u>Attitudes towards parenting</u><br>Staff could see positive aspects of relationship and parenthood for individuals with ID. They agreed that individuals with ID might be capable of having children, but they might need more support from family members or society. Also discussed they are more controlled and supervised than other parents. They noted that if a woman with ID gets pregnant, it could be viewed as a failure of the staff.

Study citation. Year. Country	Subtheme	Aim of the study	Type of study and design. Instrument and measures.	Includes participants with ID	Sample*	Main findings
**Winarni et al. (2018) Indonesia [36]**	T. Sexuality	Elucidate the attitudes towards sexuality and reproductive right in individuals with ID.	Quantitative<br><br>ASQ-ID Questionnaire	No	Proxies<br><br>both male and female<br><br>-<br><br>n=30 (100%)	Attitudes are still conservative. Attitudes towards sexual right was the highest compared to other subscales. Non-reproductive sexual behaviour and self-control attitudes were found more negative. The statements related to cultural norms and religious values were more likely related with negative attitudes. Religion and gender stand out as strong predictors in attitudes towards sexuality in ID.

* Note about Sample. First line: kind of sample (people with ID or proxies). Second line: gender. Third line: age range. Fourth line: n = participants with ID or proxies (percentage of the total sample).
"- "symbol means information in this line is unknown.

Table S2. THEME TWO. INTIMATE RELATIONSHIPS (n=30)

Study citation. Year. Country	Subtheme	Aim of the study	Type of study and design. Instruments and measures	Includes participants with ID	Sample*	Main findings
**Bane et al.. (2012) Ireland [37]**	Desires	Explore the perspectives of people with LD on relationships and supports in the Republic of Ireland.	Qualitative<br><br>Focus groups	Yes	People with ID<br><br>both; mostly female<br><br><30-30+<br><br>n=97 (100%)	People talked about loving one another. Importance of dating and being romantic. Some expressed that they did not have a relationship but would like one. One reason people found it hard to have relationships was that, in general, they felt they were treated like children. Others expressed that they would like to get married and felt it was up to themselves if they wanted to get married.
**Bates et al. (2017) UK [38]**	Desires	Focus on the intimate heterosexual relationships of adults with ID.	Qualitative Hermeneutic phenomenology<br><br>In depth interviews	Yes	People with ID<br><br>both; mostly male<br><br>35-60+<br><br>n=11 (100%)	People with ID expressed desire for a kind partner to share experiences. Having a partner suggested a sense of normality and being an ordinary member of society. People with ID might require more support to expand their social networks and to become more integrated into society.
**Bates et al. (2017) UK [39]**	Desires	Examine the importance of love to people with LD in relationships.	Qualitative Hermeneutic phenomenology analysis<br><br>Interviews	Yes	People with ID<br><br>both male and female<br><br><35-60+<br><br>n=11 (100%)	Love was important to participants and being in a romantic relationship, appeared a basic need. Relationships were viewed as a mechanism to meet certain needs such as feeling loved, company, support, intimacy and enabling people to marry. Participants saw affection, not necessarily sex, fundamental to their relationship. Participant's narratives highlighted the important role of support staff to finding and maintaining love.
**Donnachie et al. (2021) UK [40]**	Desires	Explore if people with and without ID consider themselves as desirable to others.	Qualitative<br><br>Interview	Yes	Adults with ID<br><br>both male and female<br><br>16-40<br><br>n=29 (50%)	The findings show an association between what men and women with ID and those without ID considered attractive in romantic partners. Only those with ID discussed 'companionship' as a reason for being asked out.

Study citation. Year. Country	Subtheme	Aim of the study	Type of study and design. Instrument and measures.	Includes participants with ID	Sample*	Main findings
**Giesbers et al. (2019) The Netherlands [41]**	Desires	Develop a better understanding of the unique experiences, challenges and needs of adults with mild ID with regard to their support.	Qualitative Interpretive Phenomenological Analysis (IPA)<br><br>Semi-structured interview	Yes	People with mild ID<br><br>both; mostly male<br><br>22-30<br><br>n=6 (100%)	Up to 50% of individuals with ID are chronically lonely. They want to have more friendships and/or an intimate relationship. The results also showed that relationships with staff played a more central role in the lives of participants when they had few other friendships or close relationships.
**Mattila et al. (2017) Finland [42]**	Desires	Explore how people with ID perceive love and bring out their own way of defining love and its meaning, from their own perspective.	Qualitative Theory-based qualitative content analysing method<br><br>Theme interview	Yes	Young adults with mild ID<br><br>both male and female<br><br>18-31<br><br>n=7 (100%)	Love was described by people with ID and the following themes were identified: (1) love as emotions; (2) love as acts; 3) love as knowledge and skills. Love was considered important and valuable to themselves. It was recognized as a crucial element of well-being. Love was defined even as the prerequisite of good life. It was also mentioned to be every human being's right. When thinking about the future, every participant hoped they would have love in their lives.
**O'Shea & Frawley. (2020) Australia [43]**	Desires	Explore how do contemporary young WWID relate to ideas and experiences of lives lived within discourses of gender and ID.	Qualitative Thematic analysis informed by the work of Foucault<br><br>Narrative, ethnographic and Photo-Voice methods Narrative stories	Yes	Women with ID<br><br>female<br><br>18-30<br><br>n=6 (100%)	Findings highlighted the importance relationships have in achieving a good life. However, strong echoes of the innocent, 'eternal child' discourse still remain. While parents' actions in this regard may be about safety or skills, they can also be motivated by attempts to control their offspring's sexuality and reproduction.
**Abbott & Burns. (2007) UK [44]**	Barriers and facilitators	Find out more about what helped and hindered people with ID in expressing their sexuality, meeting other LGBT people, and forming relationships.	Qualitative Constant comparative approach<br><br>Semi-structured topic guided interviews	Yes	People with ID<br><br>both; mostly male<br><br>22-59<br><br>n=20 (100%)	Relationships were important to everyone we interviewed, and people routinely had clear ideas on what they wanted from them. On the whole, LGBT people with ID were very reluctant to come out to their families, their friends, and their support staff. Many of the interviewees said that much of the name-calling and prejudice came from close family members, a situation that people found especially distressing. Unfortunately, people were more likely to have experienced more negative responses and lack of from staff and services than positive.

Study citation. Year. Country	Subtheme	Aim of the study	Type of study and design. Instrument and measures.	Includes participants with ID	Sample*	Main findings
**Bane et al. (2012) Ireland [37]**	Barriers and facilitators	Explore the perspectives of people with LD on relationships and supports in the Republic of Ireland.	Qualitative<br><br>Focus groups	Yes	People with ID<br><br>both; mostly female<br><br><30-30+<br><br>n=97 (100%)	Some people felt that it was important to have staff to talk to about relationship problems. People also said that families had a role to play, however some people explained their parents were sometimes too strict. People also talked about other things that were important in supporting them to have and keep friends, boyfriends and girlfriends: access to public transport or having their own accommodation.
**Bates et al. (2020) UK [45]**	Barriers and facilitators	Explore the views and practices of UK support staff to help people with IDD to develop loving and lasting relationships.	Qualitative<br>Latent thematic analysis<br><br>Focus group<br>Interview guide with semi-structured questions	No	Staff members supporting adults with IDD<br><br>both male and female<br><br>-<br><br>n=26 (100%)	Organisational barriers were identified: (1) Restrictive staff attitudes; (2) Lack of guidance and support for staff. Only a minority of participants had received training in sexuality and/ or relationships. The majority of staff were unsure if their organisation had a policy on sexuality and/or relationships. Lack of training and polices left staff uncertain of how they should support people regarding relationships, and as suggested above, reluctant to take any risks.
**Bates et al. (2021) UK [46]**	Barriers and facilitators	Explore how adults with IDD can be supported to form loving relationships from the perspective of family carers.	Qualitative<br>Latent thematic analysis<br><br>Focus group<br>Semi-structured open-ended questions	No	Family carers<br><br>both male and female<br><br>15-56<br><br>n=19 (100%)	Participants came up with a clear message: their relatives with IDD wanted a relationship (desire for a "normal" life), however issues related to their disabilities can make relationships challenging (impact of disability). Barriers and facilitators were identified.
**Bates et al. (2017) UK [47]**	Barriers and facilitators	Understand some of the barriers people with LD experience with regards to relationships.	Qualitative<br>Hermeneutic phenomenology<br><br>Interviews	Yes	People with LD<br><br>-<br><br>-<br><br>n=11 (100%)	The majority of professionals think barriers exist that prevent people with LD from having relationships. (1) Accommodation: participants were excluded from making life-altering choices such as with whom they lived. (2) Sexual relationships and risk avoidance: the couple's sexual relationship could be considered "highly supervised".

Study citation. Year. Country	Subtheme	Aim of the study	Type of study and design. Instrument and measures.	Includes participants with ID	Sample*	Main findings
**Callus et al. (2019) Malta [48]**	Barriers and facilitators	How people with ID experience overprotection in different aspects of life, especially through the actions of their parents.	Qualitative Social model understanding of disability<br><br>Focus groups (three rounds)	Yes	People with ID<br><br>both male and female<br><br>18-55<br><br>n=17 (34,7%)	Although some parents actively support their children's relationships, they are very often not taken seriously. Barriers generated mainly by the families were identified. Parents are right in wanting to safeguard the well-being of the adult with ID; but the pattern that consistently comes into view is of adults with ID being held back from being included in society in the name of keeping them safe from harm.
**Cytowska & Zierkiewicz. (2020) Poland [49]**	Barriers and facilitators	Learn how women with ID experience life in contemporary Polish society as gendered persons and participants of rehabilitation centres.	Qualitative Interpretative paradigm - constructivist grounded theory<br><br>Focus group Interview	Yes	Women with mild-to-moderate ID<br><br>female<br><br>19-34<br><br>n=20 (100%)	Not everyone is content with the acquaintanceships they have established. The greatest challenge facing every adult is achieving self-determination. Normally, no one surrounding their environment believes that they are capable of taking care of themselves or being responsible for the consequences of their actions. The common perception of persons with ID as "eternal children" becomes a self-replicating "social practice".
**Darragh et al. (2017) Australia [50]**	Barriers and facilitators	Explore if people with ID access internet and they use it to form relationships that express their sexuality.	Qualitative Interpretative Phenomenological Approach (IPA)<br><br>Open ended interviews	Yes	People with ID<br><br>both male and female<br><br>20-60+<br><br>n=30 (100%)	Internet was used by people with ID to explore and express their sexuality and intimacy. People with ID are capable of navigating the online world safely, this means that certain societal beliefs and attitudes do not resonate with these findings of this study.
**Deffew et al. (2022) Ireland [22]**	Barriers and facilitators	Investigate staff views and attitudes considering the impact of variables such as gender, age, education levels, religious beliefs and previous training on these attitudes.	Quantitative	No	Staff<br><br>both; mostly female<br><br>18-65+<br><br>n=86 (100%)	Very little has changed for staff supporting people with an ID in relation to sexuality and relationships. Staff generally supported people with an ID to engage in relationships and noted that it was an essential aspect of the person's rights. However, they reflected on their conflicting views on this issue concerning the person's level of intellectual disability, with staff stating their specific concerns about people with severe and profound intellectual disability.
**Friedman. (2021) USA [51]**	Barriers and facilitators	Explore the impact of the COVID-19 pandemic on the QOL outcomes of PWIDD.	Quantitative<br><br>Analysis of secondary Personal Outcome Measures (POM) QOL interviews	Yes	People with ID<br><br>both; mostly male<br><br>43 average<br><br>n=2,284 (100%)	PWID were more isolated as a result of the pandemic. PWID also frequently lack privacy, including for romantic relationships and sex; they likely had even fewer opportunities for privacy The decrease in intimate relationships among PWID is especially problematic as social and intimate relationships benefit mental health, community engagement, and QOL

Study citation. Year. Country	Subtheme	Aim of the study	Type of study and design. Instrument and measures.	Includes participants with ID	Sample*	Main findings
**Giesbers et al. (2019) The Netherlands [41]**	Barriers and facilitators	Develop a better understanding of the unique experiences, challenges and needs of adults with mild ID with regard to their support.	Qualitative Interpretive Phenomenological Analysis (IPA)<br><br>Semi-structured interview	Yes	People with mild ID<br><br>both; mostly male<br><br>22-30<br><br>n=6 (100%)	Participants talked about their experiences of stigma related to the fact that they receive support. Some felt irritated towards staff who did not allow them the chance to be as independent as possible, they explained that some staff were too quick to take over tasks from the and that they should not underestimate the abilities of people with ID.
**Lines et al. (2021) UK [52]**	Barriers and facilitators	Explore how support workers understand their role in facilitating Internet access for intimate relationships.	Qualitative Braun and Clarke's six-stage approach<br><br>Face to face interview	No	Support workers<br><br>both male and female<br><br>20-38<br><br>n=8 (100%)	Support workers said that building intimate relationships was important for adults with ID. However, there were individual dilemmas about Internet use when making decisions on how much they believed this to be part of their role. These dilemmas can become barriers in the support they provide. They were classified in: (1). social and organizational dilemmas; (2) power and position; (3) political dilemmas
**McCarthy et al. (2020) UK [53]**	Barriers and facilitators	Explore whether and how specialist dating agencies worked to support people with ID to form and maintain relationships.	Qualitative Thematic analysis<br><br>Semi-structured in-depth interview	No	Workers in specialist dating agencies<br><br>female<br><br>-<br><br>n=10 (100%)	All dating agencies shared the same aims and objectives: reduce social isolation by providing safe opportunities for people with ID to meet others for friendship and romance. Safety was high priority. All agencies reported a lower number of female members. Women's vulnerability to abuse increased their fear of being potential victims during the dating process. The views of people with ID on mainstream dating were universally negative, however most of the sample spoke very positively about specialised dating agencies.
**Pfister et al. (2020) Switzerland [54]**	Barriers and facilitators	What barriers and facilitators concerning participation are in the contexts of work, housing, education, family, relationships, and leisure time, and how do persons with physical, cognitive, and/or psychiatric impairments deal with them?	Qualitative Grounded theory<br><br>Problem-centered interview (loosely structured in-depth interview)	Yes	People with ID<br><br>mostly male<br><br>30-50<br><br>n=11 (48%)	Many interviewees reported that building a love relationship, living in a relationship, and fulfilling their sexual needs was challenging in everyday life. They viewed their participation in the life area of relationships as restricted. Disabilities create strong barriers and disadvantages when it comes to relationships The interviewees criticized that their sexual needs were not being recognized by society and important reference persons. Sexuality was a taboo subject from a social, family, and personal point of view. Families were identified as a potential barrier as in some cases.

Study citation. Year. Country	Subtheme	Aim of the study	Type of study and design. Instrument and measures.	Includes participants with ID	Sample*	Main findings
**Retznik et al. (2022) Germany [55]**	Barriers and facilitators	Examine how primary caregivers describe intimate relationships of individuals with ID.	Qualitative Summary and structuring content analysis<br><br>Semi-structured guide Interviews Focus group	Yes	People with mild-to-moderate ID<br><br>both; mostly male<br><br>14-25<br><br>n=42 (100%)	Caregivers need and want more training to support individuals with ID in forming and maintaining romantic relationships. Some caregivers do not see the young people as cognitively and psychologically able to sustain a stable, healthy and equal partnership due to their lack of mental and emotional maturity, dependency, immobility, and high care needs. All respondents stated that the partner of the young people also have a disability.
**Schaafsma et al. (2017) The Netherlands [56]**	Barriers and facilitators	Establish the perspectives of people with ID on sexuality-related topics.	Qualitative Three step approach<br><br>Semi-structured interview	Yes	People with ID<br><br>both male and female<br><br>15-52<br><br>n=20 (100%)	In previous relationships the environment had a large negative influence. Participants find having a relationship important, mainly because they do not want to be alone. They want someone to share their life with so that they can be there for one another
**Clawson. (2016) UK [57]**	Marriage	Argue that forced marriage is an issue which needs to be addressed by Safeguarding Adult Boards.	Mixed method<br><br>Survey Interview	No	Safeguarding adult boards<br><br>-<br><br>-<br><br>n=129 (100%)	There is a lack of understanding of forced marriage among frontline workers and commissioners. Adequate guidelines and materials are available, but they are not incorporated into local government and NHS policies and strategic plans.
**Clawson et al. (2020) UK [58]**	Marriage	Compare the UK demographics of forced marriage of people with LD and people without LD.	Quantitative<br><br>FMU data 2009-2015	Yes	People with LD<br><br>both male and female<br><br>11-60+<br><br>-	Risk of forced marriage is higher for people with LD. Main reason to perform them id the desire on the part of (ageing) parents to secure a reliable carer for their son or daughter. Around half of all forced marriages take place when the victim is aged between 16 and 21. Forced marriage alludes to it being an issue affecting predominantly young females, however recent data has showed men with LD are equally like to be forced to marry.

Study citation. Year. Country	Subtheme	Aim of the study	Type of study and design. Instrument and measures.	Includes participants with ID	Sample*	Main findings
**McMahon et al. (2019) UK [59]**	Marriage	Examine profiles of employment, marital status and housing between adults with and without ID in Jersey, Channel Islands.	Quantitative<br>Jersey Opinions and Lifestyle Survey	Yes	People with mild, moderate, severe and profound ID<br>both; mostly male<br>18-84<br>n=217 (8.45%)	The employment, marital status and housing profiles of adults with ID are very different compared to the general population sample. Very few adults with ID lived-in owner-occupied accommodation, this may potentially limit them on having intimate relationships.
**Pan & Ye (2012) China [60]**	Marriage	Investigate the seemingly perverted marital situation of women with ID in a village of rural China.	Mixed method<br>Case studies<br>Narrated stories<br>Survey	No	Non-random villagers sampling<br>-<br>-<br>n=60 (100%)	Marriage for women with ID in rural China is primarily for sex and having children. Carelessness and indifference are the usual characteristics of such marriages. Inadequate sex education and social services, careless family supervision increase the risks of sexual abuse of women with ID. Parents tend to support those marriages as a means of alternative caregiving.
**Savage & McConnell. (2016) Canada [61]**	Marriage	Investigate the association between disability and marital status within a large and representative sample of Canadian women.	Quantitative<br>Secondary analysis cross-sectional data Canadian Community Health Survey (CCHS).	Yes	Women with ID<br>female<br>18-59<br>n=424 (1%)	Canadian women with ID will likely enter marriage at some point. However, notably, disabled women with cognitive impairment were more than twice as likely as non-disabled women to report being 'single: separated/ divorced/widowed' and 50% of disabled women with ID were single in their fifties.
**Björnsdóttir et al. (2017) Iceland [62]**	Violence/abuse	Address the manifestation of masculinity, femininity, and autonomy in the lives of Icelanders with ID.	Qualitative<br>Thematic analysis approach<br>Interview<br>Participant observation	Yes	People with ID<br>both male and female<br>26-66<br>n=29 (65%)	About half of the women who participated in this research had experienced sexual abuse or harassment. The women who had been sexually harassed or abused seldom received any support or counselling following the incident and family members and staff commonly viewed them as incapable of experiencing trauma because of their impairment.
**Malihi et al. (2021) New Zealand [63]**	Violence/abuse	Determine the prevalence rates of non-partner physical and sexual violence in people with disabilities compared with people without these disabilities	Qualitative<br>WHO Multi-Country Study on Violence Against Women<br>Face-to-face interviews	Yes	People with ID<br>both male and female<br>-<br>n=73 (5,55%)	People with ID were more likely to report non-partner physical and sexual violence experiences. For all women with ID, parents and other family members were the main perpetrators of physical violence. Strangers were the main perpetrators for both physical and sexual violence against men with ID.

Study citation. Year. Country	Subtheme	Aim of the study	Type of study and design. Instrument and measures.	Includes participants with ID	Sample*	Main findings
**Weiss et al. (2011) UK [64]**	Violence/abuse	Examine the interaction between intellectual functioning and attachment style.	Qualitative Hierarchical regression analyses KBIT, CTQ, CADRI Questionnaire	Yes	Children with borderline-to-mild ID<br>-<br>15<br>n=40 (25%)	Experiencing maltreatment is one of the strongest predictors of violence victimization and perpetrations in dating relationships. Adolescents with borderline-to-mild ID may be more susceptible to perpetuating a cycle of violence. Adolescents with borderline-to-mild ID reported significantly more victimization and perpetration of relationship violence.
**Ward et al. (2013) USA [65]**	Intervention	Examine the outcomes of a friendship and dating program for adults with IDD.	Quantitative Baseline, post, and 10-week follow-up SNM and INI Questionnaire	Yes	People with ID<br>both male and female<br>over 18<br>n=31 (100%)	The FDP is based on the idea that safety training alone is not enough to prevent interpersonal violence. It appears participants. The results suggest the participants acquired the skills necessary to expand their social relationships.

* Note about Sample. First line: kind of sample (people with ID or proxies). Second line: gender. Third line: age range. Fourth line: n = participants with ID or proxies (percentage of the total sample).
"- "symbol means information in this line is unknown.

Table S3. SEXUAL AND REPRODUCTIVE HEALTH (n=47)

Study citation. Year. Country	Subtheme	Aim of the study	Type of study and design. Instrument and measure	Includes participants with ID	Sample*	Main findings
**Agaronnik et al. (2020) USA [66]**	Barriers and facilitators	Explore perceptions of caring for individuals with ID.	Qualitative Conventional content analysis<br><br>Open ended interviews	No	Physicians<br><br>both male and female<br><br>35-76<br><br>n=42 (100%)	Attitudes that compromise reproductive rights for women with ID persist in our society, including in health care. Barriers were identified: (1) communication; (2) routine preventive care.
**Castell & Kroese. (2016) UK [67]**	Barriers and facilitators	Develop an understanding of midwives' experiences of caring for women with a LD.	Qualitative Interpretive Phenomenological Analysis (IPA)<br><br>Semi-structured interview schedule developed in consultation with a qualified midwife Interviews	No	Midwives<br><br>female<br><br>20-50<br><br>n=9 (100%)	A number of barriers were identified with the midwives: (1) support available but not accessible; (2) having to do other people's job; (3) not skilled enough; (4) not enough time; (5) lack of LD training. Enablers were identified too: (1) focusing on build a relationship with the women and gain their trust; (2) never question the fact that women with LD are pregnant or want to become parents; (3) a positive attitude towards the women was expressed.
**Crabb et al. (2020) USA [68]**	Barriers and facilitators	Focus on two components of female reproductive health: contraception/family planning and STIs.	Quantitative<br><br>Cross-sectional survey design Medicaid managed care (MMC) enrollees Survey	Yes	Women with ID<br><br>female<br><br>53 average<br><br>n=135 (23.1%)	Women with ID were significantly more likely to talk about preventing STIs Age, race and ethnicity were significantly related to having discussions with health care providers about planning for a baby/birth control and preventing STIs.
**David et al. (1976) USA [69]**	Barriers and facilitators	Summarize a 1975 experimental program to provide family planning services mental retardation women.	Qualitative<br><br>Interview	Yes	People with mild to severe ID<br><br>both male and female<br><br>14-37<br><br>n=35 (32.11%)	The experience carried out concluded with some recommendations that would facilitate the performance of sexual and reproductive health education.
**Dotson et al. (2003) USA [70]**	Barriers and facilitators	Assess women's level of knowledge, their access to resources and their feelings of control over choices concerning their bodies.	Qualitative<br><br>Health and sexuality interviews Interview	Yes	Women with mild-to-moderate ID<br><br>female<br><br>32-40<br><br>n=8 (100%)	Most participants reported that their doctors explain things in ways they could not understand. Participants staff indicates that reproductive health is a low priority for caregivers.

Study citation. Year. Country	Subtheme	Aim of the study	Type of study and design. Instrument and measures.	Includes participants with ID	Sample*	Main findings
**Höglund & Larsson. (2019) Sweden [71]**	Barriers and facilitators	Gain a deeper understanding of midwives' perceptions of sexual health and contraceptive use of women with ID.	Qualitative<br>Content analysis<br><br>Focus group interviews with a semi-structured topic guide with open-ended questions	No	Midwives<br><br>female<br><br>-<br><br>n=19 (100%)	Midwives cited some of the barriers they have encountered in the attention to women with ID: limited information they had about women's partners and their living conditions; difficulties in identifying the level of woman's ID. They also shared some of the actions that helped them to improve their attention: extend the appointment time; adapted individual counselling according to the woman's ability and needs; they used pedagogical methods.
**Jensen et al. (2013) USA [72]**	Barriers and facilitators	Evaluate how successful primary care physicians in an academic medical centre were in providing age-, gender-, and Down syndrome-specific preventive.	Quantitative<br><br>Cohort university hospital's Central Data Repository	Yes	Women with ID (Down Syndrome)<br><br>female<br><br>18-45<br><br>n=62 (100%)	The reasons behind the inconsistent screening patterns observed in this study were identified as potential barriers: (1) poor documentation of issues discussed during a clinic visit, (2) lack of awareness by the provider of existing recommendations, (3) or more urgent medical concerns that supersede primary care discussions.
**Lee et al. (2015) Philippines [73]**	Barriers and facilitators	Contribute to the evidence available to SRH policy-makers and programs in the Philippines and elsewhere.	Qualitative<br>Thematic and data driven analysis<br><br>W-DARE<br>Face-to-face in-depth interviews<br>Focus groups	No	Service providers<br><br>both; mostly female<br><br>-<br><br>n=32 (100%)	The perspectives and experiences of service providers described reveal a range of barriers to SRH information and services for women with ID. Their perceptions of disability and linkage of the right to, and need for, SRH services with marital status were main barriers. They also reported that communication difficulties and families play as barriers in accessing to SRH services.
**Lin et al. (2010) Turkey [74]**	Barriers and facilitators	Identify and evaluate the factors regarding the utilization of the Pap smears in women with ID.	Quantitative<br>cross-sectional survey<br>2009 National Survey on Preventive Health Use and Determinants among People with Disabilities''	Yes	Women with mild, moderate and severe ID<br><br>female<br><br>33 average<br><br>n=3,283 (100%)	Predisposing factors – marital status of women with ID, and need factors – tubal ligation experience were significantly correlated to their screening uses. Mean age of the first screening was nearly 40 years old and women with ID who had a cervical smear test most often experienced pain and difficulty with the procedure.
**Lin et al. (2011) Taiwan [75]**	Barriers and facilitators	Describe caregivers' awareness of reproductive health issues with respect to women with ID who are being cared for in welfare institutions.	Quantitative<br><br>Cross-sectional, questionnaire-based study<br>"Caregiver Perceptions and Health Education Strategies with respect to Menopause WWID"	No	Caregivers<br><br>both male and female<br><br>20-66<br><br>n=1,152 (100%)	Most of the caregivers were familiar with sex education, issues of menopause, and preventive health services, but they were unfamiliar with issues concerning menstruation in women with ID. The respondent's gender, educational level and job category had a statistically significant effect on RH awareness scores.

Study citation. Year. Country	Subtheme	Aim of the study	Type of study and design. Instrument and measures.	Includes participants with ID	Sample*	Main findings
**Mac-Seing et al. (2020) Uganda [76]**	Barriers and facilitators	Explore people with ID perceptions' of possible inequities related to SRH service utilisation.	Qualitative<br>Thematic analysis<br><br>Multiple "instrumental" case study design, In-depth semi-structured interviews<br>Focus groups	Yes	People with ID<br><br>both; mostly female<br><br>-<br><br>n=7 (22%)	Barriers and facilitators were identified. Barriers: multiple intersections; experiences of discrimination and accessibility barriers. Facilitators: education opportunities and community participation and tangible policy measures.
**Mesiäislehto et al. (2021) Tanzania [77]**	Barriers and facilitators	Increase the understanding of access to SRH services of adolescent females with disabilities in Tanzania.	Qualitative<br>Empathy-based stories method (MEBS)<br><br>Verbal storytelling	Yes	Women with ID (subgroup)<br><br>female<br><br>10-19<br><br>n= 13 (9.55%)	Results showed that the SRH services did not respond well to the characteristics of the adolescent females with Barriers and facilitators were identified.
**Parish et al. (2018) USA [78]**	Barriers and facilitators	Determine the relative prevalence of discharges for reproductive cancer treatment hospitalizations linked to women with and without IDD.	Quantitative<br><br>2010 Healthcare Cost and Utilization Project (HCUP; Agency for Healthcare Research and Quality, n.d.),	Yes	Women with ID<br><br>female<br><br>61 average<br><br>n=456 (0.29%)	Women with ID were significantly younger, more likely to have public insurance coverage, more likely to live in low-income neighbourhoods, and have longer hospital stays. Women with IDD who have breast cancer are often diagnosed at an advanced stage and the delayed diagnosis often results in worse prognosis. Despite these risks, women with ID are significantly less likely than women without IDD to receive regular Pap tests or mammograms.
**Shiwakoti et al. (2021) Nepal [79]**	Barriers and facilitators	Assess the utilization of SRH services among women with disabilities and explore factors affecting it in Ilam district, Nepal.	Mixed method<br><br>Questionnaire<br>Focus group	Yes	Women with mild to profound ID (subgroup)<br><br>female<br><br>15-49<br><br>n=35 (9.1%)	Age, type of family, family size, marital status, educational status of the study population and their caretaker were significantly associated with the utilization of SRH services. Women with disabilities faced socioeconomic barriers, structural barriers and attitudinal barriers to access of SRH services.
**Sumskiene & Orlova. (2015) Lithuania [80]**	Barriers and facilitators	Explore knowledge in the field of sexuality of women WWID residing in closed care institutions in Post-Soviet countries.	Qualitative<br>Framework analysis<br><br>Expert interviews	No	Experts<br><br>both; mostly female<br><br>-<br><br>n=5 (100%)	Most of the actors (legislators and politicians; providers of mental health care services; civil society; the general public; and family members and friends) prefer not to discuss the sexual and reproductive rights of WWID residents of institutions. There are several reasons for such an attitude: deep-rooted stigma directed toward people with ID, a slow generational shift from the institutional model to a community care model.

Study citation. Year. Country	Subtheme	Aim of the study	Type of study and design. Instrument and measures.	Includes participants with ID	Sample*	Main findings
**Tanabe et al. (2015) Kenya Nepal Uganda (USA) [81]**	Barriers and facilitators	Explore the risks, needs, and barriers for refugees with disabilities to access SRH services.	Qualitative<br>Participatory activities Focus group	Yes	People with mild ID<br>both male and female<br>15-19<br>n= 51 (17.77%)	Barriers and facilitators were found. Barriers: negative and disrespectful provider attitudes were reported as the most influential barrier; communication with providers; caregiver and community attitudes. Facilitators: training providers on respectful communication skills; expanding SRH awareness activities.
**Taouk et al. (2018) USA [82]**	Barriers and facilitators	Document current awareness, attitudes, and training regarding the care of women with disabilities ob-gyns and explore barriers.	Quantitative<br>Questionnaire	No	Ob-gyns<br>both; mostly female<br>54 average<br>n=308 (100%)	Findings highlight barriers perceived by ob-gyns, as well as educational and structural needs: fear of causing patients discomfort, pain, or embarrassment; inadequate knowledge about specific disabilities and special needs; uncertainty regarding appropriate sexual and reproductive recommendations; difficulty communicating.
**Thompson et al. (2008) UK [83]**	Barriers and facilitators	Assess the sexual health promotion activities within the primary care setting across one Health and Social Services Board in NI.	Mixed method<br>Semi-structured interviews Questionnaire survey	No	GP's and PN's<br>both male and female<br>-<br>n=172 (100%)	Results from this study indicated that sexual health was most likely to be discussed with people with LD only if specifically requested. The main barrier was PN's perceived lack of knowledge/training. Issues relating to sexuality and language/ethnicity were also reported, as barriers to sexual health promotion by both GPs and PNs.
**Wickström et al. (2020) Sweden [35]**	Barriers and facilitators	Gain a deeper understanding of staff 's experiences and perceptions regarding sexual and reproductive health and rights (SRHR) related to individuals with ID.	Qualitative Content analysis with a conventional or inductive approach<br>Focus group	No	Staff that worked with people with mild-to-moderate ID<br>both male and female<br>18-65<br>n=20 (100%)	Barriers and facilitators were identified. Barriers: (1) not receiving SRH from early age; (2) very engaged parents can make issues and situations regarding sexuality complex; (3) cultural and religious differences. Facilitators: staff talked about the importance of respecting individuals with ID and encouraging them to talk to each other.
**Wiseman & Ferrie. (2020) UK [84]**	Barriers and facilitators	WWID want and would benefit from life-long, accessible, and equitable sexual and reproductive health education.	Mixed method Questionnaire Focus group	Yes	Women with ID<br>female<br>18-78<br>n=33 (100%)	Participants reported that their parents felt that having ID was in opposition to informed sexual citizenship. Differential health outcomes for WWID are a result of systemic structural inequality. Barriers to RSHP education emerged. Participants reported having limited access. The sex education they received focused exclusively on avoiding pregnancy. Participants reported a lack of formative education on menstruation. Male GPs were often cited as barriers.

Study citation. Year. Country	Subtheme	Aim of the study	Type of study and design. Instrument and measures.	Includes participants with ID	Sample*	Main findings
**Yates et al. (2021) Australia [85]**	Barriers and facilitators	Explore possible gendered barriers to applying for and receiving adequate support through the NDIS.	Qualitative<br>Reflexive thematic analysis<br><br>Interviews (semi-structured video or audio	Yes	Women with ID (subgroup)<br>female<br>20-60<br>n=7 (16,66%)	Participants had perceived differences between men and women's experiences of accessing disability support. Women are (or are expected to be) more passive and patient. Women's symptoms are often dismissed and disbelieved. Participants perceived gendered barriers to the NDIS related to motherhood, childcare and other caring responsibilities.
**Yen et al. (2014) Taiwan [86]**	Barriers and facilitators	Examine the use of Pap smear tests among women with ID in Taiwan, and the relevant influential factors.	Quantitative<br>Pap smear test data (2008)	Yes	Women with ID<br>female<br>30-70+<br>n=18,204 (100%)	WWID exhibited a significantly lower Pap test use rate. Married women with ID exhibited a higher Pap smear test usage and people with severe ID exhibited the lowest Pap smear test usage rate. Regarding age, the odds of Pap smear test usage tended to decline with increases in age.
**Goli et al. (2020) Iran [87]**	MHM (barriers and facilitators)	Determine the experiences of parents regarding the sexual and reproductive health of educable ID adolescent girls.	Qualitative<br>Conventional qualitative content analysis<br>Semi-structured interview<br>Focus group<br>Field notes	Yes	Girls with ID<br>female<br>14-18<br>n=4 (7.7%)	Parents are not sufficiently aware of the natural course of sexual development. It seems that due to the taboo associated with sexuality, parents do not have sufficient knowledge and skills required to manage the sexual behaviours of the adolescents, and they have no interest in receiving education in this regard.
**Griffin et al. (1994) Australia [88]**	MHM	Illustrate the results of the intervention.	Qualitative<br>Telephone interview	Yes	Women with severe-to-profound ID<br>female<br>25-38<br>n=30 (100%)	For of the women demonstrated significant improvement in their ability to assist their menstrual. Improvement in menstrual care skills occurred as a result of a consultative and collaborative approach. The proposed model of intervention was based, in part, on Kielhofner's model of human occupation.
**Joshi & Joshi. (2015) India [89]**	MHM	Describe the menstrual pattern and learn about problems faced by adolescent girls with varied types of disabilities in a residential institution.	Quantitative<br>Descriptive study	Yes	Women with ID<br>female<br>10-19<br>n=21 (20%)	The girls with mild ID were independent; in comparison, those with moderate ID required supervision and severe ID were fully dependent on their caretakers for maintaining menstrual hygiene. Irregular bleeding and mood swings are quite common among all girls Girls with moderate ID may be trained for independent menstrual care.

Study citation. Year. Country	Subtheme	Aim of the study	Type of study and design. Instrument and measures.	Includes participants with ID	Sample*	Main findings
**Karthikayini & Arun. (2021) India [90]**	MHM	Explore primary caretaker's challenges in managing menstruation of their adolescent girl with ID.	Qualitative<br><br>Cross-sectional study<br>In-depth interviews	Yes	Adolescent girls with moderate to severe ID<br><br>female<br><br>11-19<br><br>n=73 (100%)	This inability to manage menstruation by the ID adolescents was found to be statistically significant in comparison with the degree of ID. The respondents felt solely responsible for the menstrual cycle of their daughter. Challenges faced by caregivers were identified in relation with MHM.
**Nurkhairulnisa et al. (2018) Malaysia [91]**	MHM	Examine the menstrual pattern of girls with ID and also to evaluate the impact of menses on these girls and their parents/guardians.	Mixed method<br><br>Questionnaire<br>Interview	Yes	Women with mild, moderate and severe ID<br><br>female<br><br>9-17<br><br>n=123 (100%)	The majority of the parents/guardians have not been given adequate information regarding the menstrual care of girls with ID. Social taboo and cultural and religious believes may be the factors that prevent parents to ask for help from doctors. Parents/guardians with lower family income were significantly more aware of getting help regarding menstrual suppression.
**Pikora et al. (2014) Australia [92]**	MHM	Examine the prevalence of medical conditions and health service use among adolescents and young adults with Down syndrome.	Quantitative<br><br>Western Australian (WA) Down Syndrome population database<br>Questionnaire	Yes	People with ID<br><br>both; mostly male<br><br>16-31<br><br>n=197 (100%)	Menstrual issues have important impacts upon daily activities including restrictions on employment and social participation, as well as issues related to hygiene needs among young women with DS. Cited menstrual problems were no more common in this group than in the general population, but may be experienced more negatively and not always appropriately recognised.
**Wilbur et al. (2021) Nepal [93]**	MHM	Investigate barriers to MHM that people with disabilities and their carers face in the Kavrepalanchok, Nepal.	Qualitative<br>Phenomenological research methodology<br><br>In depth interviews<br>Observation	Yes	Women with ID<br><br>female<br><br>15-24<br><br>n=8 ("-"%)	The barriers to MHM differ depending on the person's impairment. For people with ID there may be a third party (carer) involved, who also requires MHM information and support. Such interventions must cover all aspects of MHM, including addressing harmful social beliefs.
**Wilbur et al. (2019) Nepal [94]**	MHM (intervention)	Assess the feasibility of the Bishesta campaign in the Kavre district, Nepal.	Mixed method<br><br>Pre-Post Questionnaire<br>Interview<br>Observation	Yes	Young adolescents with ID<br><br>female<br><br>15-24<br><br>n=10 (43.47%)	Bishesta campaign is acceptable and feasible. MHM information was accessible for the young people and carers with limited literacy levels. After the intervention, young people have a greater understanding that menstruation can cause discomfort and carers reported having an increased understanding of pre-menstrual and menstrual symptoms.

Study citation. Year. Country	Subtheme	Aim of the study	Type of study and design. Instrument and measures.	Includes participants with ID	Sample*	Main findings
**David et al. (1976) USA [69]**	Contraceptive choices	Summarize a 1975 experimental program to provide family planning services mental retardation women.	Qualitative<br><br>Interview	Yes	People with mild to severe ID<br><br>both male and female<br><br>14-37<br><br>n=35 (32.11%)	Very few men or women had adequate knowledge about fertility regulation or "family planning". Most people who are classified as mildly or moderately mentally retarded are capable of understanding conception, reproduction, and contraception.
**Dotson et al. (2003) USA [70]**	Contraceptive choices	Assess women's level of knowledge, their access to resources and their feelings of control over choices concerning their bodies.	Qualitative<br><br>Health and sexuality interviews<br>Interview	Yes	Women with mild-to-moderate ID<br><br>female<br><br>32-40<br><br>n=8 (100%)	Most participants indicated they had varying levels of control regarding their sexuality and reproductive health decision (no control; staff controlled these decisions; had never heard of birth control).
**Höglund & Larsson. (2019) Sweden [71]**	Contraceptive choices	Gain a deeper understanding of midwives' perceptions of sexual health and contraceptive use of women with ID.	Qualitative<br>Content analysis<br><br>Focus group interviews with a semi-structured topic guide with open-ended questions	No	Midwives<br><br>(female)<br><br>-<br><br>n=19 (100%)	Midwives considered access to contraception to be a human right and an essential factor in reproductive health for women with ID. They felt that culture and religion added to the multifaceted challenge in contraceptive counselling. WWID often had little or no knowledge about their own body and existing contraceptive methods. Woman's financial situation could also play a significant role in her contraceptive choices. Midwives' overall strategy was to be as neutral and clear as possible in providing information and counselling.
**Ledger et al. (2016) UK [95]**	Contraceptive choices	Explore who decides about contraception for women with LD.	Quantitative<br><br>Survey	Yes	Women with mild to profound LD<br><br>female<br><br>-<br><br>n=90 (100%)<br>n=21 high support needs (23.33%)	Results showed that final decisions are made by GPs followed by mothers. Women with LD usually begin to take contraception when they are not sexually active to prevent pregnancies (related to fear of abuse) and to manage menstruation. By far the most widely used form of contraception was a contraceptive implant.
**McCarthy. (2009) UK [96]**	Contraceptive choices	Investigate whether, and to what extent, women with LD were enabled to exercise choice and control when it came to their use of contraception.	Qualitative<br>Multistaged narrative analysis<br><br>Semi-structured in-depth interviews	Yes	Women with mild to moderate LD<br><br>female<br><br>20-51<br><br>n=23 (100%)	The most salient feature of the participants' responses regarding their use of contraception was a lack of autonomy. Factors contributing to this were a lack of knowledge about contraception and reproduction; most of the women lacked basic knowledge. None of the women had been given any accessible information about contraception Most reported that the decisions were made by GPs, staff in learning disability services, and parents.

Study citation. Year. Country	Subtheme	Aim of the study	Type of study and design. Instrument and measures.	Includes participants with ID	Sample*	Main findings
**McCarthy. (2011) UK [97]**	Contraceptive choices	Explore the prescribing practices and attitudes of GPs when women with ID come to them for contraception.	Mixed-method<br><br>Cross-sectional postal questionnaire survey Questionnaire-survey Individual interview	Yes	Women with ID<br>female<br>20-51<br>n=23 (100%)	GPs cited prevention of an unwanted pregnancy as the usual reason. The most commonly prescribed methods of contraception were the Pill and the Depo-Provera injection, followed by the intrauterine device (IUD). The majority of WWID were accompanied to consultations by another person. The doctors almost uniformly considered WWID to have less understanding about their contraceptive choices than women in the general.
**Melvin. (2004) UK [98]**	Contraceptive choices	Expert express their views on a real-life ethical dilemma.	Qualitative<br><br>Case study - clinical scenario Interview	No	Proxies<br>both male and female<br>-<br>n=5 (100%)	Different points of view were expressed within this case. Issues of consent, previous sex and relationships education so they can make fully informed choices were highlighted. On the other hand, mothers participating were absolutely unsupportive with this case, saying they will try to discourage it at all costs and being sure their influence is strong enough to persuade their acceptance of contraception.
**Schwartz et al. (2020) USA [99]**	Contraceptive choices	Describe the characteristics and experiences of levonorgestrel IUD use in nulliparous children, adolescents, and young adults with disabilities.	Quantitative<br><br>Retrospective cohort review	Yes	Women with ID<br>female<br>≤22<br>n=115 (62%)	Very few participants were sexually active when IUD was inserted. The majority of IUD placements occurred at least one year after menarche. Almost all were placed on the operating room and nearly half of them were inserted at the time of another procedure. The number of patients with follow-up declined over time.
**Taouk et al. (2018) USA [82]**	Contraceptive choices	Document current awareness, attitudes, and training regarding the care of women with disabilities ob-gyns and explore barriers.	Quantitative<br><br>Questionnaire	No	Ob-gyns<br>both; mostly female<br>54 average<br>n=308 (100%)	Barriers for contraceptive choice were identified: (1) ability to utilize contraception; (2) decision-making capacities for contraception and sex; (3) consent to irreversible means of contraception; (4) patient understanding of contraception risks and benefits; (5) patient understanding of STD or pregnancy prevention.
**Walmsley et al. (2016) UK [100]**	Contraceptive choices	Root the reproductive experiences of women with LD within the context of wider debates on human rights, reproductive justice and supported decision-making.	Qualitative<br><br>Interviews	Yes	Women with ID<br>female<br>-<br>n=19 (100%)	Women with LD are insufficiently well informed about sexual relationships, contraception and the type to use to make an informed choice about using it. Most reported having the decision made for them. We found that the use of the contraceptive pill or implant was primarily to regulate periods and reduce the pain and discomfort associated with the menstrual cycle.

Study citation. Year. Country	Subtheme	Aim of the study	Type of study and design. Instrument and measures.	Includes participants with ID	Sample*	Main findings
**Wickström et al. (2020) Sweden [35]**	Contraceptive choices	Gain a deeper understanding of staff 's experiences and perceptions regarding sexual and reproductive health and rights (SRHR) related to individuals with ID.	Qualitative<br>Focus group	No	Staff that worked with people with mild-to-moderate ID<br>both male and female<br>18-65<br>n=20 (100%)	Staff agreed that the initiative to discuss contraception often comes from parents and from staff, more rarely from the individuals themselves, especially if he/she has a more severe form of ID. Many WWID use contraception to reduce menstrual bleeding, but pregnancy-prevention should also be mentioned. Contraceptive implants or injections are the most commonly used methods among individuals with ID as they make things easier for the staff.
**Wiseman & Ferrie. (2020) UK [84]**	Contraceptive choices	WWID want and would benefit from life-long, accessible, and equitable sexual and reproductive health education.	Mixed method<br>Questionnaire<br>Focus group	Yes	Women with ID<br>female<br>18-78<br>n=33 (100%)	Women felt that they had minimal information and knowledge about the range of contraceptives available. As a result, women's ability to make choices about contraception and family planning were not only restricted but actively guided towards hormonal contraceptive measures. Decisions were made on two bases: either to suppress menstruation or to avoid pregnancy risk.
**Wu et al. (2018) USA [101]**	Contraceptive choices	Estimate provision of moderately and highly effective reversible contraceptives to women IDD.	Quantitative<br>Massachusetts All-Payer Claims Database	Yes	Women with IDD<br>female<br>15-44<br>n=13,059 (1.43%)	Women with IDD were more likely to use LARC. Caregivers, including family members and residential facility staff play an important role in contraceptive selection and access. There is evidence that caregivers request contraception on behalf of women with IDD for no contraceptive reasons.
**Agaronnik et al. (2020) USA [66]**	Sterilisation	Explore perceptions of caring for individuals with ID.	Qualitative<br>Conventional content analysis<br>Open ended interviews	No	Physicians<br>both male and female<br>35-76<br>n=42 (100%)	Several participants expressed strong views regarding routine contraception and sterilization for women with ID. OB/GYNs described sterilization of patients with ID as a routine practice and offered different rationales.
**Björnsdóttir et al. (2017) Iceland [62]**	Sterilisation	Address the manifestation of masculinity, femininity, and autonomy in the lives of Icelanders with ID.	Qualitative<br>Interview<br>Participant observation	Yes	People with ID<br>both male and female<br>26-66<br>n=29 (65%)	All of the participants had been told directly or indirectly that they were not capable of being mothers. Sterilization of PWID in Iceland is gendered and also sexualized as practices have been justified as a sexual abuse prevention.

Study citation. Year. Country	Subtheme	Aim of the study	Type of study and design. Instrument and measures.	Includes participants with ID	Sample*	Main findings
**Christian et al. (2001) USA [21]**	Sterilisation	Determine the attitudes and knowledge of support staff at an agency serving individuals with IDD.	Quantitative<br>Survey	No	Staff<br>both; mostly female<br>+18<br>n=43 (100%)	Most caregivers support the rights of women with disabilities. However, they do not agree with the idea that women with ID should not have children because they have a disability or that the degree of severity should be taken into account, nor do they agree that sterilization is a supported and approved contraceptive method for women with ID.
**Hamilton. (2015) New Zealand [102]**	Sterilisation	Report the views of non-medical interest groups about sterilization and people with ID in New Zealand.	Quantitative<br>Survey	No	Proxies<br>both; mostly female<br>18-60<br>n=67 (100%)	The majority of participants indicated that a procedure might be warranted for both men and women with ID within certain circumstances. For men with ID: lack of control over sexual responses and individual choice. For women with ID, managing sexual desire, fertility control, unwanted pregnancy and vulnerability to abuse.
**Li et al. (2018) USA [103]**	Sterilisation	Compare overall female sterilization, hysterectomies and age of sterilization in three mutually exclusive groups.	Quantitative<br>Secondary analysis of data from the National Survey of Family Growth (NSFG)<br>Interviews via computer-assisted personal	Yes	Women with ID<br>female<br>15-44<br>n=1,308 (13.1%)	Findings revealed that women with cognitive disabilities had the greatest risk of receiving any type of sterilisation. Sterilised women with ID were the youngest, had less education, and were most likely to have income below the Federal Poverty Level and less likely to be married.
**Márquez-González & Valdez-Martínez. (2018) Mexico [104]**	Sterilisation	Favour reflection on an archetypal ethical problem (non-therapeutic hysterectomy in adolescents with ID) that paediatric clinicians face in their clinical practice.	Qualitative<br>Case study	Yes	Women with severe and moderate ID<br>female<br>12-13<br>n=13 (100%)	The predominance of physicians' biological approach, leaves aside the fact that adolescents with ID are biopsychosocial units. Is usual to find that the potential results of hysterectomy are usually presented to the parents in terms of "better quality of life". There would be no doubt that the parents would accept the hysterectomy.
**Márquez-González, et al. (2021). Mexico [105]**	Sterilisation	Determine the frequency of hysterectomy and the clinical and epidemiologic characteristics that associated to the indication of hysterectomy in girls and young females with ID.	Quantitative<br>A cross-sectional, descriptive, chart review study<br>Questionnaire	Yes	Women with moderate to profound ID<br>female<br>≤25<br>n=50 (100%)	Non-therapeutic hysterectomy continues to be one common procedure performed in females with ID. Cultures where contradicting a physician is considered disrespectful parents/carers may assume a "passive patient "role. Also, presenting hysterectomy as a safe surgical procedure and having preventive benefits, clearly might influence in the parent's choice.
**Aderemi et al. (2014) South Africa [106]**	HIV/STIs	Investigate HIV testing prevalence and factors associated with the utilization of HIV VCT among PWID.	Quantitative<br>Survey	Yes	People with ID<br>both male and female<br>15-49<br>n=81 (19.7%)	HIV testing prevalence is lower among PWID than in the general population, with non-significant gender difference. PWID are less likely to utilize VCT services.

Study citation. Year. Country	Subtheme	Aim of the study	Type of study and design. Instrument and measures.	Includes participants with ID	Sample*	Main findings
**Aderemi et al. (2013) Nigeria [107]**	HIV/STIs	This study documents the levels of HIV knowledge and sexual practices among LMID compared with those of NDL in Nigeria.	Quantitative<br><br>Comparative, cross-sectional study<br>Questionnaire	Yes	People with mild-to-moderate ID<br><br>both male and female<br><br>12-19<br><br>n=257 (50%)	*Barriers*<br>Persons with ID have inadequate exposures to HIV information. Male with ID reported better access than female with ID to HIV information. Radio and television were the main sources; however, they present HIV messages in formats that are not always accessible to PWID.<br>*Knowledge*<br>Having ID was significantly associated with lower HIV transmission knowledge.
**Schenk et al. (2020) USA Ghana Uganda Zambia [108]**	HIV/STIs	Explore access to and use of HIV information and services among persons with disabilities in three sub-Saharan countries.	Qualitative<br><br>Multi country exploratory situation<br>Interview<br>Focus group<br>Framework analysis approach	Yes	People with ID<br><br>both male and female<br><br>-<br><br>n=263 (21.29%)	Barriers found: misconceptions about sexual activity among persons with disabilities; lack of information; misinformation and community beliefs; literacy challenges; vulnerability to abuse; complexity of stigma across multiple layers; attitudes among service providers. Facilitators found: employing messages relevant for persons with and without disabilities promoting peer leadership.
**Schmidt et al. (2019) USA [109]**	HIV/STIs	Examine the association between I/DD and the prevalence of STIs and STI testing.	Quantitative<br><br>Cross-sectional study<br>Thompson Reuters Databases 2014–2015	Yes	People with IDD<br><br>both; mostly male<br><br>15-64<br><br>n=25,193 (50%)	Individuals with IDD had significantly lower odds of an STI diagnosis. However, the three most common STI tests were similar for individuals with and without I/DD (asymptomatic STI screening, syphilis and gonorrhoea).

* Note about Sample. First line: kind of sample (people with ID or proxies). Second line: gender. Third line: age range. Fourth line: n = participants with ID or proxies (percentage of the total sample).
"- "symbol means information in this line is unknown.

Table S4. THEME FOUR: SEXUALITY AND SEX EDUCATION (n=28)

Study citation. Year. Country	Subtheme	Aim of the study	Type of study and design. Instrument and measures	Includes participants with ID	Sample*	Main findings
**Björnsdóttir et al. (2017) Iceland [62]**	Knowledge	Address the manifestation of masculinity, femininity, and autonomy in the lives of Icelanders with ID.	Qualitative<br><br>Interview<br>Participant observation	Yes	People with ID<br><br>both male and female<br><br>26-66<br><br>n=29 (65%)	People with ID are not perceived to have the status of autonomous agents and often have limited opportunities to make decisions in everyday life. Men interventions contained traditional ideas of gender and performed hegemonic masculinity. Women performed independence and resourcefulness in their narratives. However, they also described how they were often not allowed to make decisions. Possessing personal autonomy and agency is fundamental for the development of gender and sexual identities.
**Brkić-Jovanović et al. (2021) Serbia [110]**	Knowledge	Assess the sexual activity, and their level of knowledge regards sexuality of persons with ID residing in institutional housing.	Quantitative<br><br>General Sexual Knowledge Questionnaire<br>What-if-situations-test<br>Hulbert index of sexual assertiveness<br>Questionnaire completed in the form of interviews	Yes	People with ID<br><br>both; mostly female<br><br>47 average<br><br>n=100 (100%)	The results indicate that most persons with ID who participated in the study were sexually active. These findings confirm that persons with ID have sexual needs and the capacity to engage in sexual behaviour. However, the results show an extremely low level of knowledge of sexuality and sexual intercourse, especially poor knowledge of pregnancy, contraception and sexually transmitted diseases. Participants were shown to be mostly aware of basic risk situations and that they could ask for help if they ever found themselves in them. However, their strategies for dealing with potentially dangerous situations were far from adequate.
**Christian et al. (2001) USA [21]**	Knowledge	Determine the attitudes and knowledge of support staff at an agency serving individuals with IDD.	Quantitative<br>Survey	No	Staff<br><br>both; mostly female<br><br>+18<br><br>n=43 (100%)	Most respondents agreed that women with IDD should be given the opportunity to receive sex education. More than half said they would feel comfortable implementing such training, however less than one-third said they had received training to do so. Most staff did not appear to rely on the policy to guide their responses. These findings suggest that although most staff would ensure that the woman in this scenario received sex education, they were guided more by their personal views, experiences and histories than by the agency's sexuality policy.
**Frawley & Wilson. (2016) Australia [111]**	Knowledge	Explore sexual education among a small group of young adults with ID who were involved in transition to adulthood employment support programs in Australia.	Qualitative<br>Constant comparison method of grounded theory<br><br>Focus groups with a semi-structured interview guide with open-ended questions	Yes	People with ID<br><br>both; mostly male<br><br>17-20<br><br>n=25 (100%)	Accessible sex education and information is needed for young people with ID. Young men knowledge about sex came mainly from pornography and magazines. Family is a key source of information for young men. Young women knew that the contraception prevented pregnancy but could not describe how, and for most the use of contraceptive methods was other's decision. Hygiene and menstruation were the focus of most information they had received mainly from mothers. Sex education that had been provided at school was not seen as very useful.

Study citation. Year. Country	Subtheme	Aim of the study	Type of study and design. Instrument and measures.	Includes participants with ID	Sample*	Main findings
**Isler et al. (2009) Turkey [112]**	Knowledge	Examine the knowledge, opinions, attitudes and concerns of the parents regarding sexuality of their children with ID.	Quantitative<br>Post session 1 and 2 Questionnaire	No	Parents of children with mild-to-moderate ID<br>both male and female<br>34-69<br>n=40 (100%)	It has been observed that the majority of the parents have not been professionally trained on the subject of sexuality. Almost half of the parents have said that they have not talked about sexuality with their children. While half of the parents stated in the study that sexual education should start during the elementary school years, they have started providing sexual education to their own children during high school.
**Isler et al. (2009) Turkey [113]**	Knowledge	Determine characteristics of the development of adolescent knowledge, opinions, and attitudes regarding sexuality in adolescents with mental disability.	Qualitative<br>Face to face questionnaire	Yes	People with mild-to-moderate ID<br>both; mostly male<br>15-20<br>n=60 (100%)	Half of the participants stated that they did not receive any education on sexuality. Adolescents exhibited a very low level of knowledge, there was difficulty in identifying gender-specific differences in reproductive organs. It has been suggested that sexual education should start in the family and continue at school as a formal sexual education program, supported by health care professionals. Students with ID want information about male and female reproductive organs, anatomy, and physiology of the reproductive system
**Olaleye et al. (2007) Nigeria [114]**	Knowledge	The paper seeks to explore sexual behaviours and reproductive health knowledge among in-school young people with disabilities (PWD) in Ibadan, Nigeria.	Quantitative<br>Questionnaire	Yes	People with ID<br>both; mostly female<br>10-25<br>n=10 (9.70%)	Respondents with ID were the most sexually active, but they possessed the least knowledge about contraceptives (and condoms), HIV/AIDS and source of reproductive health services. The vulnerability of persons with disabilities to sexual abuse was confirmed in the study. The most vulnerable group were those with ID, as half of them had been raped while young people. Many young people ID considered sexual abuse as a sign of acceptance and were unable to recognise what constitute an abuse.
**Pownall et al. (2020) UK [115]**	Knowledge	Explore the impact of both intellectual ability and the extent of social networks. To achieve this, we compared health knowledge across three groups of adolescents.	Quantitative<br>Multi-choice questionnaire with visual illustrations	Yes	People with mild ID<br>both male and female<br>18 average<br>n=29 (35%)	There were significant differences between the groups' knowledge of pregnancy and contraception. The most commonly held misconceptions by young with ID included the beliefs that sexual intercourse hade to occur more than once for someone to become pregnant and that the contraceptive pill prevented HIV. They were unsure if the condom could prevent STIs. Sources of information may also have contributed to different pattern of health knowledge across the groups of participants

Study citation. Year. Country	Subtheme	Aim of the study	Type of study and design. Instrument and measures.	Includes participants with ID	Sample*	Main findings
**Schaafsma et al. (2017) The Netherlands [56]**	Knowledge	Establish the perspectives of people with ID on sexuality-related topics.	Qualitative<br>Three step approach<br><br>Semi-structured interview	Yes	People with ID<br><br>both male and female<br><br>15-52<br><br>n=20 (100%)	Of the 20 participants 19 report to have received sex education: 6 of the young adults at school, others from a staff member, a parent or by reading a book. The older adults from a parent, teacher or a course. All participants received sex education once or twice during their life. Topics mentioned do not cover the entire area of sexuality Participants indicated that getting sex education was interesting, fun and nice; however, they also mentioned class members being a bit giggly or ashamed and acted a bit tough.
**Thompson et al. (2016) Australia [116]**	Knowledge	Examine clinicians' perspective on the usefulness and usability of the exiting sex education tools.	Qualitative<br>Content analysis<br><br>Face-to-face semi-structured interviews	No	Clinicians who worked directly with PWID<br><br>both; mostly female<br><br>-<br><br>n=23 (100%)	Clinicians reported that sexual knowledge assessment tools provided them with a useful foundation to base their work. Available sexual knowledge assessment tools for people with ID are essentially similar in structure and content. Overall, issues such as body parts and menstruation were generally addressed well, but topics such as sexual dysfunction and sexual aids received little or no coverage in any tool The ASK is the only tool to contain a Problematic Socio-Sexual Behaviours Checklist. With dates of publication between 1994 and 2006, it is unsurprising that no tool included content related to current information technology such as sex and the internet and sexting. A clear need for the tools to be brought up to date is needed.
**Girgin-Büyükbayraktar et al. (2017) Turkey [117]**	Barriers and facilitators	Determine what sexual problems that individuals with special educations needs have and how to provide sexual education for these students, depending on the opinions of the teachers of mentally handicapped individuals.	Qualitative<br>Content analysis technique<br><br>Semi-structured interview	No	Teachers<br><br>both male and female<br><br>25-40<br><br>n=25 (100%)	There is a lack of information about suppressing sexual feelings of individuals with ID and punishing those which cannot be suppressed. During sexual education, the smallest details and individual differences should be taken into consideration. Barriers and facilitators were identified. Barriers: development of wrong attitudes, parents' failure to cooperate, generalizing, habits and withdrawal. Facilitators: individual differences, cooperation with parents, suppressing sexual feelings, choosing appropriate environments, sanitation rules and receiving information from a same-sex specialist.
**Gürol et al. (2014) Turkey [118]**	Barriers and facilitators	Evaluate the views of the mothers having children with ID regarding providing sexual education for their children and protect them against possible dangers by creating sufficient awareness on this subject.	Qualitative<br>Content analysis method<br><br>Focus group Interviews	Yes	Girls with ID<br><br>female<br><br>7-18<br><br>n=9 (100%)	All mothers who participated in this study stated that they did not provide sexual education for their children with ID. Due to the traditional family structure of the Turkish culture, sexual education and numerous definitions about the word sexual are still considered taboo and talking about sexual information, especially with girls. Mothers commonly stated that children with intellectual disability could not and should not have a sex life.

Study citation. Year. Country	Subtheme	Aim of the study	Type of study and design. Instrument and measures.	Includes participants with ID	Sample*	Main findings
**Hanass-Hancock et al. (2018) South Africa [119]**	Barriers and facilitators	Investigate the contextual factors that enabled or provided barriers for implementation of comprehensive sexuality education with learners with ID.	Mixed method<br><br>Pre-intervention and post-implementation cross-sectional survey<br>Survey<br>In-depth interviews (educators)<br>Focus group (learners	No	Educators<br><br>both; mostly female<br><br>-<br><br>n=12 (100%)	Cultural norms, combined with religious beliefs were powerful personal barriers identified by educators. Some of the trained educators were able to overcome personal barriers. Age, cultural upbringing and a lack of sexuality education training were identified as significant barriers for their peers. Some staff members showed particular discomfort with some topics. Younger educators perceived themselves as more flexible and able to challenge thinking, social beliefs and norms. All reported that the school environment was a crucial contextual factor that could function as an enabler or barrier.
**Löfgren-Mårtenson. (2009) Sweden [120]**	Barriers and facilitators	Identify, describe and understand the opportunities and hindrances for young people with ID in expressing a variety of sexual expressions and examine in which way the influence of environment may impact the sexuality of young adults with ID.	Qualitative<br>Theoretical Framework (symbolic interactionism)<br><br>Interviews<br>Participant observations	Yes	People with ID<br><br>both male and female<br><br>16-27<br><br>n=13 (36%)	Not a single parent reported that their son or daughter was homosexual or had ever expressed a desire for same-sex activity; similar results were expressed by caring and service personnel. A few of these caregivers and staff members recounted that they had seen homosexual acts between individuals with ID. But they interpreted those acts as expressions of friendship, not sexual desire. The absence of bisexuals or homosexuals among young people with ID can also be seen as a result of how these young people are socialized more generally. Education in schools still tends to promote a heterosexual norm.
**Menon & Sivakami. (2019) India [121]**	Barriers and facilitators	Understand parents' perceptions and concerns about the sexuality and reproductive health of their child with an IDD.	Qualitative<br>Thematic analysis<br><br>Interview	Yes	Children with IDD<br><br>both male and female<br><br>10-30<br><br>n=7 (50%)<br><br>(*) Note:<br>Primary caregivers as informants (both male and female)	Findings highlighted that the type of disability may influence perceptions and concerns. It can be concluded that parental perceptions about the sexuality and reproductive health of their child with IDD may influence the way important life decisions of their life are taken. The study was also able to identify factors that influence parental perceptions and concerns.

Study citation. Year. Country	Subtheme	Aim of the study	Type of study and design. Instrument and measures.	Includes participants with ID	Sample*	Main findings
**Murphy et al. (2016) USA [122]**	Barriers and facilitators	Determine if genetic counsellors were being asked to provide sex education counselling on a variety of topics to patients with ID ages 9–17 and what barriers prevented them from doing so.	Quantitative<br><br>Questionnaire	No	Genetic counsellors<br><br>both male and female<br><br>-<br><br>n=60 (100%)	Responses indicated that their comfort in providing sex education is dependent upon external factors such as the level of the patient's ID and counsellor's knowledge of the patient's culture. For both age groups the barriers included: 1) not having enough time during a patient encounter; 2) lack of training; 3) the patient's ID is too profound; and 4) that genetic counsellors should not be responsible for providing sex education counselling. One barrier that was noted for the 9–12 age was that the patient was perceived to be too young.
**Nelson et al. (2020) Sweden [123]**	Barriers and facilitators	Explore what it meant for a group of teachers in southern Sweden to teach SRHR to students with ID.	Qualitative Phenomenological approach<br><br>Interview	No	Teachers<br><br>both male and female<br><br>24-55<br><br>n=10 (100%)	Findings from this study reveal that teaching SRHR to students with intellectual disabilities can be understood in terms of 'accepting the challenge to coach special-needs students into adulthood'. Barriers and facilitators were encountered.
**Pownall et al. (2012) UK [33]**	Barriers and facilitators	Explore parents' attitudes towards sex related topics discussions with their offspring with ID.	Mixed method<br><br>In-depth interview Questionnaire	Yes	Young people with mild ID<br><br>both male and female<br><br>16-24<br><br>n=30 (50%)	Mothers of young people with ID had spoken about fewer sexual topics with their offspring, began these discussions at a later age, and these tended to being less detailed. Mothers were particularly concerned about their offspring's vulnerability to exploitation and abuse. Indeed, mothers of young people with ID placed more emphasis on discussing safety issues with their child than any of the other topics.
**Wheeler. (2007) UK [124]**	Barriers and facilitators	Explore how a group of men with learning disabilities in South Wales experienced their sexuality and sexual identity.	Qualitative Interpretative Phenomenological Analysis (IPA)<br><br>Focus groups In-depth semi-structured interviews	Yes	Men with LD<br><br>male<br><br>16-42<br><br>n=12 (100%)	Barriers were identified. (1) paternalistic, dependency-inducing approach; (2) lack of work to improve self-esteem and positive body image; (3) no enough training for specialists; (4) prejudices and stereotypes of some members of society; (5) hostility from formal and informal carers and general public; (6) inaccurate information.

Study citation. Year. Country	Subtheme	Aim of the study	Type of study and design. Instrument and measures.	Includes participants with ID	Sample*	Main findings
**Baines et al. (2018) UK [125]**	Sexual intercourse	Explore sexual activity and sexual health amongst young people with mild/moderate ID.	Qualitative<br><br>Secondary analysis Waves Interview	Yes	People with mild-to-moderate ID<br><br>both male and female<br><br>-<br><br>n=527 (3.60%)	Young people with mild/moderate ID were more likely to have experienced a range of social and material disadvantages Peer victimisation was also significantly higher. They experienced more episodes of violence. Both men and women with ID who were bullied were more likely to report unsafe sex on >50% of occasions. People with ID were less likely to have had sexual intercourse by age 19/20 than their peers. However, if they were sexually active then: (1) girls with ID were significantly less likely to have had their first experience of sexual intercourse below the age of 16; (2) boys and girls with ID were significantly more likely to commonly have unsafe sex; (3) girls with ID were more likely to have been pregnant; and (4) were more likely to be mothers.
**Cheng & Udry. (2005) USA [126]**	Sexual intercourse	Examine the sexual behaviours of adolescents with low cognitive abilities as compared to those of the mentally average.	Quantitative<br><br>Survey	Yes	People with ID<br><br>both; mostly female<br><br>15-16<br><br>n=21 (5%)	Compared to adolescents with average intelligence, higher percentages of the young with ID have not experienced romantic attraction to either sex or do not know their sexual preferences. Overall, the majority of adolescents with ID have not had coital sex.
**Shandra & Chowdhury. (2012) USA [127]**	Sexual intercourse	Examine the relationship between having a disability and type of relationship with an adolescent's first sexual partner, level of discussion about contraception before first sexual intercourse, and pregnancy expectation at first sexual intercourse.	Quantitative<br><br>National Longitudinal Survey of Youth 1997 (NLSY97)	Yes	Women with ID<br><br>female<br><br>12-16<br><br>n= "-" (0.04%)	Results suggest that having mild ID increases the likelihood of having first sexual intercourse with a stranger versus a steady dating partner. These results also may reflect the increased likelihood for women with ID to be victims of sexual assault. Girls with multiple conditions or severe ID who do not use contraception at first sexual intercourse are also much more likely to want a pregnancy.
**Shandra et al. (2016) USA [128]**	Sexual intercourse	Understand the relationship between disability and the context of first sexual intercourse among boys with ID.	Quantitative<br><br>The National Longitudinal Survey of Youth 1997 (NLSY97)	Yes	Men with ID<br><br>male<br><br>12-17<br><br>n= "-" (7.27%)	Those with learning disabilities are more likely to report very early sexual debut (between ages 12 and 14 years) and to talk a lot about birth control but less likely to use condoms if they do use contraceptive methods. Gender and type of disabling condition intersect to shape the context of first sexual intercourse. Evaluation of training resources for the sexual education of people with moderate to severe LD found a lack of suitable materials and assessments. our results indicate the importance of providing boys with LD with accessible sexual knowledge and the tools with which to discuss and make informed decisions around that knowledge.

Study citation. Year. Country	Subtheme	Aim of the study	Type of study and design. Instrument and measures.	Includes participants with ID	Sample*	Main findings
**Box & Shawe. (2014) UK [129]**	Intervention	Explore participants with ID experiences of attending a sexuality and relationship group.	Qualitative Qualitative content analysis<br><br>Case study methodology following a qualitative theoretical perspective Participant observation Semi-structured interviews	Yes	People with mild-to-moderate ID<br><br>both; mostly male<br><br>20-49<br><br>n=5 (100%)	Participants who had a lower pre-group score achieved a larger number of correct answers in their post-group scores. Participants generally had better knowledge of social aspects, and tended to not score well in the sexual knowledge sections. Participants' general experience of the group was positive, they would like the group was facilitated by a male and female together. Participants generally preferred having the sex education sessions before the relationship sessions, as this helped them to have a knowledge base of sexuality topics and terminology used.
**De Mello et al. (2021) Brazil Spain [130]**	Intervention	Analyse DFGs transfer and impact on adolescent girls with ID.	Qualitative Communicative methodology (exclusionary and transformative dimension)<br><br>Interview Field diary analysis Focus group	Yes	Women with mild-to-moderate ID<br><br>female<br><br>15-24<br><br>n=32 (100%)	The results of the case study offer insights based on two central ideas: (1) the transferability of DFGs as a space for dialogic interactions to the context of special education and (2) evidence of the impact that DFGs are having on the lives of adolescent girls with intellectual disabilities, specifically, how they are promoting preventive interactions that can contribute to protecting these girls from gender-based violence relationships.
**Goli et al. (2021) Iran [131]**	Intervention	Compare the effect of two educational interventions on mothers' awareness, attitude and self-efficacy about the sexual health care of ID adolescent girls.	Quantitative<br><br>Cluster randomized control trial (2 intervention groups, 1 control group) Pre-post Questionnaire	No	Mothers of educable intellectual disabled adolescent girls<br><br>female<br><br>-<br><br>n=81 (100%)	Major barriers about sexual health education for adolescent daughters identified by the mothers were their own insufficient knowledge about sexual issues, embarrassment surrounding discussions of this issue with their daughters, fear of the arrogance and curiosity of girls, and a lack of skills for effective communication. The results showed that the mean score of mothers' awareness, attitude and self-efficacy in each of the groups after the intervention was significantly different from their scores before the intervention. The results even showed that one month after the intervention, the mean score higher in the "group training".
**Gutiérrez-Bermejo, et al. (2021) Spain [132]**	Intervention	Analyse the sexual experience, behaviours, and attitudes towards sexual relations of adults with ID and implement an intervention program and obtain pre and post data on attitudes towards responsible sexual relationships.	Quantitative<br><br>Pre-experimental Pre-post Questionnaire	Yes	People with mild to severe ID<br><br>both male and female<br><br>22-67<br><br>n=44 (100%)	This empirical study offers evidence demonstrating the usefulness of a brief intervention program to improve the knowledge and attitudes toward consensual and responsible sexual relationships in people with disabilities. The questionnaire measured the cognitive, affective and conative components of sexual behaviour. The results obtained with the program showed effectiveness in improving the various components of responsible attitudes toward sexuality.

Study citation. Year. Country	Subtheme	Aim of the study	Type of study and design. Instrument and measures.	Includes participants with ID	Sample*	Main findings
**Randell et al. (2021) Sweden 133]**	Intervention	Explore an intervention using the Toolkit "Children what does it involve?" and the Real-Care-Baby (RCB) simulator among students with ID.	Qualitative<br>Qualitative content analysis<br><br>Post-intervention Toolkit "Children what does…" RCB (Real Care Baby) Simulator<br>Interview	Yes	Teenagers with mild to moderate ID<br>both male and female<br>16-20<br>n=16 (100%)	After the experience, the students described the educational material in favourable terms. They felt they learned many new things about the consequences of having a child in relation to time, money, skills, relationship and housing. Results show that students generally voiced appropriate and realistic expectations about parenthood and that the combination of theoretical knowledge and practical experiences helped the participants to grasp the notion of parenting/parenthood.

* Note about Sample. First line: kind of sample (people with ID or proxies). Second line: gender. Third line: age range. Fourth line: n = participants with ID or proxies (percentage of the total sample).
"- "symbol means information in this line is unknown.

Table S5. THEME FIVE: PREGNANCY (n=32)

Study citation. Year. Country	Aim of the study	Type of study and design. Instrument and measure	Includes participants with ID	Sample*	Compared to	Main findings
**Akobirshoev et al. (2019) USA [134]**	Investigate racial and ethnic disparities in birth outcomes and labour and delivery-related charges among women with IDD.	Quantitative<br><br>Secondary analysis Hierarchical mixed-effect logistic and linear regression models Healthcare Cost and Utilization Project National Inpatient Sample	Yes	Women with ID<br><br><25-35+<br><br>n=2,110 (100%)		We found marked racial and ethnic disparities in stillbirths within the population of women with IDD. The proportion of Black and Hispanic women with IDD who had a stillbirth was almost twice as high. People who have ID and are also members of racial/ethnic minority groups, in general, face greater barriers in healthcare access and receive lower quality services. Hispanic adults with IDD were also more likely to be obese and have diabetes and were more likely to experience a range of challenges in accessing health care, including lack of knowledge of the health system, being unsatisfied with services, not having services available in the area, lack of transportation, high costs of services and language barriers.
**Akobirshoev et al. (2017) USA [135]**	Explore the birth outcomes of infants compared to the general obstetric population.	Quantitative<br><br>Nationwide Inpatient Sample (NIS) of the Health Care and Cost Utilization Project (HCUP),	Yes	Women with ID<br><br><25-34+<br><br>n=1,897 (0.04%)	Women without ID	Women with IDD who delivered were more likely to be Black and to come from lower income households, they were more likely to be aged <25 years and to have one or more comorbidities. Women with IDD had higher risk of having preterm births, low births, and stillbirths.
**Andrews et al. (2021) USA [136]**	Qualitatively explore the lived experiences of disabled women related to breastfeeding.	Qualitative Traditional descriptive content analysis procedures<br><br>Semi-structured interview	Yes	Women with IDD<br><br>18-60<br><br>n=3 (13%)		Barriers and facilitators were identified. Barriers: 1) communication difficulties with lactation consultants, 2) intense pressure to breastfeed, 3) milk supply and latch problems. These barriers are systemic and may require community-and-society-level intervention. Facilitators: (1) positive interactions with health care providers: disability-affirmative lactation education, a fair and balanced presentation of options without shaming women for either choice.
**Bacharach & Baumeister. (1998) USA [137]**	Test the influence of maternal IQ on cognitive growth among at-risk LBW children.	Quantitative<br><br>Multi-site randomized clinical trial Infant Health and Development Program (IHDP) (1990).	Yes	Women with ID<br><br>-<br><br>-		The variable that stands out as the major contributor to child IQ among premature LBW children is maternal IQ. Results of our analyses go beyond confirming the well-established correlation between maternal and child intelligence. But we add to this picture that family income is a somewhat more potent mediator than home environment on effects of maternal IQ.

Study citation. Year. Country	Aim of the study	Type of study and design. Instrument and measures.	Includes participants with ID	Sample*	Compared to	Main findings
**Biel et al. (2020) USA [138]**	Compare the laboured status of deliveries (laboured or unlaboured) between women with and without disabilities.	Quantitative<br><br>Retrospective cohort study hospital discharge and vital records data (birth certificates and death files) for all births in California between 2000 and 2012	Yes	Women with ID<br><br>-<br><br>n=858 (0,04%)	Women without ID and with other disabilities	Results regarded higher proportions of C-sections deliveries among women with ID. Findings suggests that deliveries among women with disabilities may sometimes be more complicated. Anticipation of potential complications during labour and vaginal delivery may contribute to the higher proportions of unlaboured deliveries. Alerting women to this fact would allow them to prepare for the possibility of an intrapartum caesarean. Findings also have identified a pattern of unlaboured caesarean deliveries that do not appear to be medically indicated.
**Bradbury-Jones et al. (2015) UK [139]**	Explore disabled women's experiences of accessing and using maternity services when they were affected by domestic abuse.	Qualitative<br>Critical Incident Technique<br><br>Critical Incident Technique Interviews	Yes	Women with mild ID<br><br>-<br><br>n=1 (20%)	Women with other disabilities	Women's narratives were dominated by fear of disclosure, and the resulting consequences of staff judgement and loss of control. For these participants, knowledge was empowering, enabling them to make their own choices and feel in control of their care decisions. Biomedical jargon excludes women from fully accessing information about their care. Lack of information or incorrect information diminished women's trust in health professionals. Their biggest concern was the desire to be seen as "normal pregnant women". Because their pregnancies were perceived be health professionals to be "abnormal", their care was dominated by the social norms of a traditional medical model, rather than those of woman-centred care. Women's experiential knowledge of pregnancy was frequently dismissed by health professionals.
**Brown et al. (2019) Canada [140]**	Document the reproductive health of women with IDD, including their fertility rates, pregnancy outcomes and reproductive health after pregnancy.	Quantitative<br><br>Health and social services administrative data	Yes	Women with IDD<br><br>-<br><br>n=3,932 ("-" %)	Women without IDD	Women with IDD were more likely to be <20 years of age and to live in the lowest-income neighbourhoods at the time of pregnancy. They were more likely to have comorbid conditions and mental health problems. Their pregnancies were more likely to end in stillbirth, to have labour inductions and C-sections. Among live births, their infants were more likely to be born preterm, to be small for gestational age, to experience neonatal morbidity and to die in the first month of life. In the first year postpartum, women with IDD were more likely to visit an emergency department and to be hospitalized. Finally, they were more likely to deliver a second baby within 12 months of the first.
**Brown et al. (2016) Canada [141]**	Compare their risks for adverse maternal and neonatal outcomes to those of women with IDD.	Quantitative<br><br>Population-based cohort between April 1, 2002, and March 31, 2012 (ICES)	Yes	Women with IDD<br><br><20-35+<br><br>n=1,852 (47.1%)	Women with other disabilities	Women with IDD, require better prevention of social and health risk factors prior to pregnancy and increased surveillance for complications during pregnancy. High rates of poverty, poor maternal health, and substance use contribute to increased risk for preterm birth and neonatal morbidity among infants.

Study citation. Year. Country	Aim of the study	Type of study and design. Instrument and measure	Includes participants with ID	Sample*	Compared to	Main findings
**Brown et al. (2016) Canada [142]**	Examine the occurrence of labour induction, caesarean section and operative vaginal delivery in women with IDD compared to those without and determine whether specific, identifiable pre-pregnancy health conditions and pregnancy complications.	Quantitative<br><br>Population-based retrospective cohort study Institute for Clinical Evaluative Sciences database	Yes	Women with ID<br><br>18-64<br><br>n=3,932 (1.01%)	Women without ID	We found that women with IDD, compared to those without, were slightly more likely to have labour inductions and caesarean sections but not operative vaginal deliveries. Pre-existing health conditions and maternal complications explained some of the elevated occurrence of labour inductions and caesarean sections, specifically psychiatric disorders and pre-eclampsia/eclampsia. There may be risk factors, including lifestyle behaviours (e.g, smoking, diet) and control of chronic health conditions (e.g, psychiatric disorders) which could be targeted.
**Brown et al. (2018) Canada [143]**	Estimate and compare the risk of discharge to child protective services directly from the birth hospitalization in newborns of women with IDDs versus without IDDs.	Quantitative<br><br>Population-based cohort Population based study of newborns	Yes	Women with IDD<br><br><20-35+<br><br>n=3,845 (1%)	Women without IDD	Women with IDD were more likely than those without IDD to be <20 years of age and to live in low-income neighbourhoods and rural areas. They were more likely to have chronic medical conditions, mental illness, and substance use disorders and to receive social assistance. Their infants were more likely to be born preterm and to have neonatal morbidity and congenital anomalies. 1 in 20 new-borns of women with IDD were discharged to child protective services immediately after the birth hospitalization. Women with IDD have a particularly high risk for custody loss.
**Brown et al. (2018) Canada [144]**	Compare the risk of rapid repeat pregnancy among women with and without IDDs.	Quantitative<br><br>Population-based cohort study Institute for Clinical Evaluative Sciences databases	Yes	Women with IDD<br><br>≤19-≥40<br><br>n=2,855 (0.30%)	Women without IDD	Women with IDD were at increased risk for rapid repeat pregnancy within 12 months of a live birth. Rapid repeat pregnancy risk was also greatest in high-risk groups defined by lower income and receipt of social assistance. This rapid repetition reflects vulnerability in a woman's ability to make informed reproductive decisions and lack of access to family planning services.
**Clements et al. (2020) USA [145]**	Examine outpatient care utilization during the postpartum period among women with IDD relative to women without IDD.	Quantitative<br><br>Matched cohort study design The Massachusetts All Payers Claims database (APCD)	Yes	Women with ID<br><br>15-44<br><br>n=962 (25%)	Women without IDD	Women with IDD were more likely to be aged <19 years, have lower household incomes, and more likely to have Medicaid insurance coverage. They were more likely to have delivered a preterm infant and to have a hospital length of stay after delivery >5 days. The most common visit type, for both groups, in the early and late postpartum periods was for interview, evaluation or consultation.
**Darney et al. (2017) USA [146]**	Describe primary C-section delivery among women with and without disabilities.	Quantitative<br><br>Retrospective cohort study California Department of Health Services, 2006	Yes	Women with ID<br><br>-<br><br>n=1,103 (0.02%)	Women without ID and with other disabilities	Women with ID they are younger, less educated, poorer, less likely to be married, and considerably less likely to be able or willing to name the baby's father on the birth certificate. Women with IDD are more likely to be obese and smoke during pregnancy, also less likely to receive prenatal care during the first trimester. Results also showed that women with ID were more likely to deliver by caesarean. Women with ID tend to experience more complications during pregnancy, such as preeclampsia.

Study citation. Year. Country	Aim of the study	Type of study and design. Instrument and measure	Includes participants with ID	Sample*	Compared to	Main findings
**Gaskin & James. (2006) UK [147]**	Investigate whether a structured interview based on a simplified form of the EPDS items, using a visual scale to depict severity of symptoms, would be more accurate that the EPDS in identifying postnatal depression in mothers with learning difficulties.	Mixed method<br><br>EPDS questionnaire Semi-structured interview	Yes	Women with mild to moderate LD<br><br>18-37<br><br>n=13 (50%)		The study reported here suggests, for mother with LD, caution should be exercised in interpreting EPDS scores as an indicator of postnatal depression. It suggests that the structured interview was a reliable indicator, as mother were reporting symptoms which people close to the ere also observing to be present.
**Gleason et al. (2021) USA [148]**	Evaluate risk of a range of obstetric interventions and adverse maternal outcomes, including severe maternal morbidities (SMM) and mortality, among women with and without disabilities.	Quantitative<br><br>Retrospective cohort, secondary analysis medical chart review for deliveries between January 2002 and January 2008.	Yes	Women with ID<br><br>29 average<br><br>n=91 (0.04%)	Women without ID and with other disabilities	Women with ID had higher risk of almost all pregnancy complications, obstetric interventions, and adverse outcomes. They often have higher risk factors for poor maternal outcomes, including living in poverty, smoking, substance use, and depression. They also face barriers to care, including financial barriers. They also report negative reactions toward their pregnancy, which extends to health care practitioners and may affect the quality of care provided and lead to refusal of care for these women.
**Ha & Martinez. (2021) USA [149]**	Determine the association between disability and self-reported infertility and explore whether disability status affects whether women seek medical attention for infertility.	Quantitative<br><br>2013–2014, 2015–2016, and 2017–2018 waves of the National Health and Nutrition Examination Survey (NHANES)	Yes	Women with ID<br><br>33 average<br><br>n=356 (9.39%)	Women without ID and with other disabilities	Having any disability was associated with increased odds of having self-reported infertility, and having disabilities appeared to be generally related to lower odds of seeking reproductive healthcare for infertility. Several categories of unmet reproductive health needs for WWID can be identified. People with ID consistently report higher rates of obesity, lack of physical activity, and smoking. These socio-behavioural risk factors could be contributing to higher risk of infertility and other reproductive complications.
**Höglund et al. (2012) Sweden [150]**	Investigate antenatal health and demographic factors as well as pregnancy and delivery outcomes in women with ID in Sweden compared to women without ID or any psychiatric diagnosis.	Quantitative<br><br>Population based data National Patient Register (NPR) and the Medical Birth Register (MBR)	Yes	Women with IDD<br><br>16-46<br><br>n=326 (0.09%)	Women without IDD	At the first antenatal visit more women with ID did not cohabit with the father of the expected child and did not work. The proportion of teenage births (11–19 years) was higher in women with ID The occurrence of obesity was higher too. After birth, women with ID were more likely to be discharged from the maternity ward directly to a place other than their homes.
**Höglund et al. (2012) Sweden [151]**	Describe mode of birth, preterm birth rates, Apgar scores, the incidence of being small for gestational age (SGA), stillbirth and overall perinatal death in children born to mothers with ID in comparison to mothers without ID or any other psychiatric diagnosis.	Quantitative<br><br>Swedish population-based sample National Patient Register and Medical Birth Register	Yes	Women with ID<br><br>-<br><br>n=326 (0.09%)	Women with other disabilities	A greater proportion of children born to mothers with ID than children born to mothers without ID were born by CS. These children also scored <7 at one and five minutes in Apgar test more often. More children of women with ID were born preterm. Stillbirth was almost four times more prevalent and perinatal death was more than four times more common among those born to mothers with ID.

Study citation. Year. Country	Aim of the study	Type of study and design. Instrument and measure	Includes participants with ID	Sample*	Compared to	Main findings
**Horner-Johnson et al. (2019) USA [152]**	Examine and describe the timing and frequency of prenatal care among women with physical, or sensory disabilities, or IDD compared with women without disabilities.	Quantitative<br><br>Retrospective cohort study	Yes	Women with IDD<br><br>16-35<br><br>n= 1,670 (0.03%)	Women without IDD	Women with IDD are less likely to receive adequate prenatal care. They are also more likely to have modifiable risk factors for adverse pregnancy outcomes (e.g., smoking, obesity). These issues speak to the need for preconception care that assesses sexual activity and pregnancy desires of women with IDD and directly addresses pregnancy planning and appropriate preparations.
**Malouf et al. (2017) UK [153]**	Investigate access and quality of maternity care for women with differing disabilities.	Quantitative<br><br>Structured cross-sectional study design secondary analysis2015 national survey of women's experience of maternity care<br>Survey	Yes	Women with ID<br><br>16-35+<br><br>n=127 (0.63%)	Women without ID and with other disabilities	All women with LD were at a higher risk for delivering preterm. They reported more negative experiences of pregnancy care, particularly in relation to always being spoken in a way that they could understand. Advice about contraception was less available. Women with LD reported that their personal circumstances had not been taken into account.
**Malouf et al. (2017) UK [154]**	Explore the lived experiences of pregnancy, childbirth, prenatal and postnatal care and services received.	Qualitative<br>Interpretative Phenomenological Analysis (IPA)<br><br>Interview	Yes	Women with mild to severe ID<br><br>25-39<br><br>n=9 (100%)		Barriers and facilitators were identified.<br>Barriers: Women with LD expressed encountering negative attitudes and denial of choice in their attention. Communication barriers were identified, written information and verbal communication with health. Participants shared the feeling of having to prove themselves in relation to their capability to become mothers.<br>Facilitators: As main facilitators, participants expressed the importance of finding positive attitudes that do not contribute to scrutiny and support for choice support.
**Mitra et al. (2019) USA [155]**	Examine the emergency department (ED) use in the postpartum period among Massachusetts mothers with IDD.	Quantitative<br><br>Massachusetts Pregnancy to Early Life Longitudinal data	Yes	Women with ID<br><br><20-39+<br><br>n=776 (0.13%)	Women without ID	Women with IDD were more likely to be younger, non-Hispanic Black or Hispanic, report fewer years of education, have public health insurance and receive less than adequate prenatal care. Were less likely to be married or to have identified a father on the birth certificate, more likely to have smoked during pregnancy, experienced 1+ pregnancy-related comorbidities, had a low birth weight infant and had a Caesarean delivery. The prevalence of any ED visit was nearly three times higher in women with IDD compared with women without IDD for all three postpartum periods.

Study citation. Year. Country	Aim of the study	Type of study and design. Instrument and measure	Includes participants with ID	Sample*	Compared to	Main findings
**Mitra et al. (2018) USA [156]**	Examine the risk of postpartum hospital admissions and emergency department (ED) visits among U.S. women with intellectual and developmental disabilities (IDD).	Quantitative<br><br>Population-based retrospective cohort study Pregnancy to Early Life Longitudinal Data System	Yes	Women with IDD<br><br><20-39+<br><br>n=1,104 (0.14%)	Women without IDD	Women with IDD were more likely to be younger, have a lower level of education, be non-Hispanic Black or Hispanic, have public health insurance, and were less likely to be married. They were more likely to smoke during pregnancy, have one or more pre-pregnancy comorbidities or delivery related complications, have a low birth weight infant, and have a C-section delivery. Women with IDD had higher prevalence rates for hospital admission and ED visits during all critical postpartum periods.
**Mitra et al. (2018) USA [157]**	Explore non-delivery antenatal hospital utilization of women with IDD compared to women without IDD.	Quantitative<br><br>Population-based retrospective cohort study Massachusetts Pregnancy to Early Life Longitudinal Data System (PELL)	Yes	Women with ID<br><br><20-40+<br><br>n=498 (24.54%)	Women without ID	Women with IDD were more likely to have antenatal hospital utilization. They face multiple challenges related to accessibility, quality, and content of prenatal care. Barriers include communication difficulties and potentially stigmatizing perceptions. The American Congress of Obstetrics and Gynaecology does not have prenatal guidelines specific to women with IDD. Information about pregnancy and childbirth is generally not accessible to women with IDD.
**Mueller et al. (2019) USA [158]**	Compare pre-pregnancy characteristics, pregnancy outcomes, and rehospitalisation <2 years after delivery among women with and without ID.	Quantitative<br><br>Population-based linked vital records-hospital discharge data (1987-2012)	Yes	Women with IDD<br><br>18-53<br><br>n=103 (9.05%)	Women without IDD	Women with ID were younger and unmarried at delivery and smoked more often. We observed that women with ID were more than twice as likely to have inadequate prenatal care, as an increased risk of gestational diabetes and preeclampsia.
**Murthy et al. (2014) India [159]**	Compare reproductive health parameters including pregnancy experience, health access during pregnancy and type of delivery among women with disability compared to women with no disability.	Quantitative<br><br>Questionnaire	Yes	Women with ID<br><br>15-45<br><br>n=11 (4.4%)	Women without ID and with other disabilities	Reproductive health experiences differed significantly between women with ID. There was a significant difference between the proportion of women with disability reporting diabetes and depression. Women with disability reported less attention during their pregnancy by health personnel.
**Parish et al. (2015) USA [160]**	Compare women with ID pregnancy outcomes and their infants' health to other mothers.	Quantitative<br><br>2010 Healthcare Cost and Utilization Project Nationwide Inpatient Sample	Yes	Women with ID<br><br><18-34<br><br>n=340 (0.04%)	Women without ID	Women with IDD were more likely than general obstetric population to have adverse pregnancy and foetal/infant outcomes. They tended to be younger, were more likely to be black and also more likely to have public health insurance coverage. They also had more negative emotional experiences through pregnancy.

Study citation. Year. Country	Aim of the study	Type of study and design. Instrument and measure	Includes participants with ID	Sample*	Compared to	Main findings
**Potvin et al. (2020) Canada [161]**	Describe pregnancy and childbirth-related knowledge among two Canadian women with IDD, the perinatal informational support they received during their pregnancies, and barriers and facilitators to obtaining this support.	Quantitative<br><br>Secondary analysis Semi-structured interview	Yes	Women with ID<br><br>20-30<br><br>n=2 (100%)		Women demonstrated childbearing-related knowledge which appeared to increase with subsequent pregnancies. They had difficulties identifying signs of pregnancy. They relied on formal sources of informational support. Prejudicial attitudes of caregivers inhibited women with IDD from disclosing their pregnancies and needs. Perinatal informational support appeared to be especially influenced by information format, the women's level of autonomy, and the nature of caregiver involvement.
**Redshaw et al. (2013) UK [162]**	Describe the maternity care provided during pregnancy, birth and the postnatal period for women with a disability and explore disabled and non-disabled women's perceptions of care received during these periods.	Quantitative<br><br>2010 Care Quality Commission (CQC) Questionnaire	Yes	Women with LD<br><br><20-35+<br><br>n=120 (8%)	Women without LD and with other disabilities	Women with LD had more contact with antenatal services during the pregnancy. They tend to have a C-section and it seem to be a planned procedure. During pregnancy, reported being spoken in a way they could not understand. During labour, their partner was hardly welcomed. Few participants reported being involved in decisions about their care.
**Rubenstein et al. (2020) USA [163]**	Replicate and expand upon past findings focused on pregnancy complications and maternal birth outcomes in women with IDD.	Quantitative<br><br>Longitudinal cohort Big Data for Little Kids (BD4LK) project from 2007–2016	Yes	Women with ID<br><br>≤18-≥40<br><br>n=1,032 (0.58%)	Women without ID	Gestational diabetes and gestational hypertension were slightly more common in women with IDD; they were more likely to be transferred to another medical facility during labour and they had 1.20 times the risk of caesarean delivery. Women with ID were less likely to receive prenatal care in the first trimester; indeed, black women with IDD were at higher risk to not receive first trimester prenatal care.
**Shin et al. (2020) South Korea [164]**	Compare the pregnancy and neonatal outcomes in women with and without disabilities and investigate the risks of pregnancy and neonatal complications among women with various types and differing levels of severity of disabilities.	Quantitative<br><br>Databases of the Korea National Health Insurance (KNHI) claims, National Health Screening Program for Infants and Children (NHSP-IC) and Disability Registration System in Korea.	Yes	Women with ID<br><br>-<br><br>-	Women without ID and with other disabilities	Our study confirms that women with disabilities have higher adverse perinatal outcomes. Women with ID had the greatest number of complications. They showed an increased risk of caesarean delivery. They also have limited or delayed medical services because they cannot recognize the signs and symptoms of pregnancy in itself or pregnancy complications.

Study citation. Year. Country	Aim of the study	Type of study and design. Instrument and measure	Includes participants with ID	Sample*	Compared to	Main findings
**Tarasoff et al. (2020) Canada [165]**	Describe the preconception health characteristics of women with disabilities compared to women without such disabilities.	Quantitative<br><br>Population-based, cross-sectional study	Yes	Women with ID<br><br>15-44<br><br>n=8,986 (0.33%)	Women without ID and with other disabilities	Across all indicators, women with disabilities had poorer preconception health. Women with IDDs were the most socioeconomically marginalized group and had the greatest health disparities. They were more likely to experience sexual and reproductive health education barriers. They are less likely to receive preventive care including cancer screening and family planning.
**Wickström et al. (2017) Sweden [166]**	Investigate whether children born to mothers with ID have an increased risk of being diagnosed with mental illness, injuries, and violence compared with children born to mothers without ID.	Quantitative<br><br>Population-based register study on a cohort of children born between 1999 and 2005 Medical Birth Register (MBR).	Yes	Women with ID<br><br>27 average<br><br>n=2,749 (0.57%)	Women without ID	Mothers with ID were younger and cohabited less often with the child's father. These mothers also had a lower level of education and less disposable mean income. A higher proportion of the mothers with ID had mental health problems and used tobacco, alcohol, and drugs before and during pregnancy. The children born to mothers with ID were more often born premature and were at a greater risk of being diagnosed with mental health problems and epilepsy in early childhood. Furthermore, children of mothers with ID had an increased risk of being exposed to injuries, violence, and child abuse. It was found that children of mothers with ID had a three times higher risk of being victims of violence and abuse

* Note about Sample. First line: characteristic of the participating women including ID severity when known. Second line: age range. Third line: n = participants with ID (percentage of the total sample).
"-" symbol means information for this line is missing.

Table S6. THEME SIX: EXPERIENCING PARENTHOOD (n=4)

Study citation. Year. Country	Subtheme	Aim of the study	Type of study and design. Instrument and measures	Includes participants with ID	Sample*	Main findings
**Collings et al. (2020) Australia [167]**	Mother's point of view (violence)	Report child removal and domestic and family violence.	Qualitative Inductive content analysis<br><br>Semi-structured interview	Yes	Women with ID<br><br>21-51<br><br>n=10 (100%)	Barriers to access of domestic violence services by women with intellectual disability were identified. Intimate partner violence jeopardised their ability to keep their own children safe, leading to child protection involvement and child removal.
**McCarthy. (2019) UK [168]**	Mother's point of view	Give insights into the lives of women with learning disabilities as they experience domestic violence.	Qualitative Interpretative Phenomenological Analysis (IPA)<br><br>In-depth interviews	Yes	Women with mild LD<br><br>23-41<br><br>n=6 (100%)	Domestic violence was there at the very start of her journey towards motherhood. Four main themes emerged from the data: becoming a mother mothering while living with domestic violence, the broad spectrum of abuse; and losing the children.
**Symonds et al. (2021) UK [169]**	Father's point of view	Understand the perspectives of fathers with LD on being a father and the support they had received.	Qualitative Thematic analysis (Braun and Clarke)<br><br>Semi-structured interviews	Yes	Men with LD (fathers)<br><br>26-61<br><br>n=10 (100%)	Themes and subthemes were identified. (1) Descriptions of fatherhood; (2) Domestic duties and fathering; (3) Challenges of fatherhood; (4) Support with fatherhood.
**Weiber et al. (2019) Sweden [170]**	Children's point of view	Describe the experience of having grown up in a family where the mother has an IDD.	Qualitative Narrative interviewing and content analysis<br><br>Retrospective narrative Interview	No	Daughters to mothers with ID<br><br>30-50<br><br>n=4 (100%)	Participants defined their childhood experiences full of abuse, neglect, deprivation, anxiety, overburdening responsibilities, and a general lack of stability and support.

* Note about Sample. First line: kind of sample (people with ID or proxies). Second line: age range. Third line: n = participants with ID or proxies (percentage of the total sample).
